# What types of objective measures have been used to assess core ADHD symptoms in children and young people in naturalistic settings? A scoping review

**DOI:** 10.1136/bmjopen-2023-080306

**Published:** 2024-09-12

**Authors:** Charlotte Rose Kelman, Jo Thompson Coon, Obioha C Ukoumunne, Darren Moore, Rebecca Gudka, Eleanor F Bryant, Abigail Russell

**Affiliations:** 1 Children and Young People's Mental Health (ChYMe) Research Collaboration, University of Exeter, Exeter, Devon, UK; 2 NIHR CLAHRC South West Peninsula (PenCLAHRC), University of Exeter Medical School, Exeter, UK; 3 University of Exeter, Exeter, UK

**Keywords:** child & adolescent psychiatry, impulse control disorders, psychometrics

## Abstract

**Abstract:**

**Objectives:**

We described the range and types of objective measures of attention-deficit/hyperactivity disorder (ADHD) in children and young people (CYP) reported in research that can be applied in naturalistic settings.

**Design:**

Scoping review using best practice methods.

**Data Sources:**

MEDLINE, APA PsycINFO, Embase, (via OVID); British Education Index, Education Resources Information Centre, Education Abstracts, Education Research Complete, Child Development and Adolescent Papers, Cumulative Index to Nursing and Allied Health Literature (CINAHL), Psychology and Behavioural Sciences Collection (via EBSCO) were searched between 1 December 2021 and 28 February 2022.

**Eligibility Criteria:**

Papers reported an objective measure of ADHD traits in CYP in naturalistic settings written in English.

**Data extraction and synthesis:**

2802 papers were identified; titles and abstracts were screened by two reviewers. 454 full-text papers were obtained and screened. 128 papers were eligible and included in the review. Data were extracted by the lead author, with 10% checked by a second team member. Descriptive statistics and narrative synthesis were used.

**Results:**

Of the 128 papers, 112 were primary studies and 16 were reviews. 87% were conducted in the USA, and only 0.8% originated from the Global South, with China as the sole representative. 83 objective measures were identified (64 observational and 19 acceleration-sensitive measures). Notably, the Behaviour Observation System for Schools (BOSS), a behavioural observation, emerged as one of the predominant measures. 59% of papers reported on aspects of the reliability of the measure (n=76). The highest inter-rater reliability was found in an unnamed measure (% agreement=1), Scope Classroom Observation Checklist (% agreement=0.989) and BOSS (% agreement=0.985). 11 papers reported on aspects of validity. 12.5% of papers reported on their method of data collection (eg, pen and paper, on an iPad). Of the 47 papers that reported observer training, 5 reported the length of time the training took ranging from 3 hours to 1 year. Despite recommendations to integrate objective measures alongside conventional assessments, use remains limited, potentially due to inconsistent psychometric properties across studies.

**Conclusions:**

Many objective measures of ADHD have been developed and described, with the majority of these being direct behavioural observations. There is a lack of reporting of psychometric properties and guidance for researchers administering these measures in practice and in future studies. Methodological transparency is needed. Encouragingly, recent papers begin to address these issues.

**Strengths and limitations of this study**

- Our scoping review is the first to identify objective measures for attention-deficit/hyperactivity disorder in naturalistic settings.
- Our scoping review has been conducted and reported according to best practice, following the PRISMA-ScR (Preferred Reporting Items for Systematic reviews and Meta-Analyses extension for Scoping Reviews) guidelines to ensure a high standard of methodological and reporting quality.
- The terms ‘objective’ and ‘naturalistic’ can be understood differently, and the criteria we have used may limit the scope of our findings.
- Searches were however conducted in 2022 and more recent papers may have advanced the field, such as the recent publication of reporting guidelines for psychometric properties.

## Introduction

### Attention-deficit hyperactivity disorder

Attention-deficit/hyperactivity disorder (ADHD) is a neurodevelopmental disorder with an estimated global prevalence of 5.29% (95% CI: 5.01 to 5.56).^1^ It is characterised by chronic hyperactivity, impulsivity and/or inattention, across multiple settings. Although traits can be found across all children and young people (CYP), they are more prominent in those with ADHD. These traits can negatively impact functioning at home, school or in social situations,^2^,^4^ and can be a risk factor for numerous functional impairments that often persist into adulthood.^5^,^7^

Despite ADHD being highly prevalent, there are still significant gaps and discord in research, particularly regarding assessment and diagnosis.^8^ An ADHD diagnosis is based on psychiatric criteria, such as the Diagnostic and Statistical Manual of Mental Disorders-5 (DSM) or the International Classification of Diseases 11, both of which require symptoms to be present prior to 12 years old,^9^ and across settings.^10 11^ With the exception of these diagnostic criteria, there is no definitive framework for assessing ADHD, with variations observed among different nations and even within specific geographical regions, each adhering to unique assessment processes. Health institutions typically follow clinical guidelines, such as The National Institute for Health and Care Excellence (NICE) guidelines,^12^ to recognise, assess and manage ADHD. Internationally, all guidelines specify who can diagnose and prescribe, and emphasise the importance of clinical interviews, direct observations, family history and recommend rating scales as auxiliary tools, however adherence is not mandatory.^13^ Due to the heterogeneous nature of ADHD, most guidelines suggest using subjective measures from multiple informants (eg, teachers, parents and clinicians) that rely on individual beliefs or perceptions to inform the outcome.^14^ However, opinions may be biased or influenced by the setting in which the informant knows the child. This subjectivity often leads to significant inconsistencies in ratings across sources, with low agreement between parent and teacher reports being typical in ADHD evaluations.^15 16^ Clinicians instead may rely on their own judgement and experience.^17^,^21^ This has led to suggestions that objective measures could alleviate some bias and improve diagnostic accuracy.

An objective measure of ADHD assesses traits through non-opinion-based means. Examples include continuous performance tests (CPTs),^22^ systematic behavioural observations,^23 24^ acceleration-sensitive tests,^25 26^ virtual reality and functional MRI.^27^ Objective measures mitigate issues such as informant bias and inconsistencies, which subjective measures are prone to. Objective measures are often not implemented in research and clinical settings, despite being recommended.^28 29^ Implementation could offer further evidence towards assessment. Previous reviews have reported that objective measures can exhibit high reliability and validity but demonstrate variability.^24 30 31^ For example, Minder *et al* found the inter-rater reliability across systematic behavioural observations ranged from 0.61 to 1 (Pearson’s r) and from 0.39 to 0.99 (kappa coefficient), and convergent validity varied across studies and tools, with correlations ranging from poor to strong. Objective measures have been found to have good discriminant validity between ADHD and neurotypical people, however, are not as effective as discriminating between ADHD and other disorders.^24 32^

ADHD symptoms must be present across all settings to meet diagnostic criteria. However, diagnostic assessments typically occur in controlled clinical settings that lack ecological validity and cannot replicate real-world experiences, and ADHD traits are dynamic.^10 11^ The nature and incidence of ADHD behaviours can vary between settings and across similar situations within one setting.^33 34^ Hence, there is a definite need to assess ADHD in the presence of those uncontrollable factors, as diagnosis is predicated on symptom presence and impairment across all settings.^35^ In turn, the clinical symptoms for ADHD are behavioural, and in settings, such as school, impairment is likely be greater and children may struggle to cope.^36^ Therefore, it is important that assessments consider environmental influences.

Several recent reviews of objective measures in ADHD have been conducted^24 29 32 37^; however, each review focused in depth on a specific type of measure rather than the breadth of what measures have been studied in the research literature. Considering the importance of both objective measures and naturalistic settings in the assessment of ADHD, it is currently unclear which objective measures of ADHD could be used in naturalistic settings. Due to the exploratory nature of this aim, a scoping review was deemed the most appropriate method for this study. A scoping review is an analysis of existing literature, focusing on the breadth and scope of a topic.^38 39^ Unlike systematic reviews, which delve deeply into specific questions, scoping reviews prioritise breadth over depth.^38^ This descriptive scoping review highlights the array of measures used in the field and what information is reported about them across studies, and identifies gaps in knowledge and informs future research.

### Objectives

We aimed to describe the types of objective measures of ADHD in CYP that have been applied in naturalistic settings and reported in research.

The research questions (RQs) were:

1. What are the existing objective measures of ADHD in CYP that could be applied in naturalistic settings?
2. What types of objective measures are there?
3. What populations have been included in this body of research?
4. What is the reliability and validity of the objective measures?
5. How were the objective measures implemented?

## Methods

### Protocol and registration

A protocol, written in line with the PRISMA-ScR guidelines,^40^ is available on the University of Exeter repository.^41^

### Eligibility criteria

#### Study design

Any study designs were eligible, including systematic reviews. Studies included within systematic reviews were used to identify objective measures.

#### Participants

CYP aged 18 years old or under presenting with any of the three main ADHD symptoms: hyperactivity, impulsivity and inattention. CYP with a clinical or research diagnosis, or presenting symptoms of ADHD as indicated by a validated measure were eligible for inclusion.

#### Outcomes

Any objective measure used in a naturalistic setting or that could be applied in a naturalistic setting to assess symptoms of ADHD, including behavioural observations, accelerometers, and rating scales. Rating scales recording perceptions of symptom severity or occurrence were excluded.

#### Settings

Naturalistic settings, defined as a child’s everyday variety of settings, included home, school or community spaces. If a study took a child out of their everyday routine, it was not eligible for inclusion, for example, summer ‘treatment’ programme settings.

#### Date and language

Papers from any country published from 1987, due to reconceptualisation from Attention Deficit Disorder (ADD) to ADHD in the DSM. Only papers written in English were included to prevent any miscommunication when translating.

### Information sources

#### Selection of sources of evidence

The search strategy aimed to identify published peer-reviewed journal papers and grey literature. An initial preliminary search of MEDLINE, EBSCO and Embase via OVID was conducted to locate papers relevant to this area and to scope the size of the evidence base.

##### Electronic databases

MEDLINE, APAPsychINFO, Embase, (via OVID); British Education Index, Education Resources Information Centre, Education Abstracts, Education Research Complete, Child Development and Adolescent Papers, Cumulative Index to Nursing and Allied Health Literature (CINAHL), Psychology and Behavioural Sciences Collection (via EBSCO) were searched between 1 December 2021 and 28th February 2022. Grey literature searching was conducted between 30 June 2022 and 31st August 2022.

### Search strategy

The search strategy can be found in the online supplemental material.

### Identification of papers for inclusion

All records were double screened. Duplications were removed and studies were imported into CADIMA for title and abstract screening which took place independently by CRK, RG and EB. These authors then conducted independent full-text screening. If necessary, discrepancies were resolved by consensus or referral to AR.

#### Data charting process

Data charting, whereby relevant data were extracted from studies, was completed using Microsoft Excel to facilitate. The draft charting spreadsheet was piloted on an initial 20 papers. After piloting, amendments were made to ensure that it captured all relevant details to answer our RQs. Data were extracted by the lead author. A random sample of 10% of papers was checked by SH (Suzie Holt) and KC (Kirsty Corwell) and as the error rates were low this was considered adequate. Data were extracted or charted for 39 features of included papers. These included key data about the study type and design, participant characteristics and detailed information on the objective measure including psychometric data. A full list of items is reported in the online supplemental material.

#### Synthesis of results

Papers were grouped and synthesised based on commonalities. In the process of reviewing the included studies, we identified recurring themes and categories that were prevalent across multiple papers. Consequently, we adopted these categories as the basis for our analytical framework. The key defining feature that grouped papers was whether objective measures were behaviour observations or acceleration-sensitive devices.

#### Patient and public involvement statement

Patient and public involvement was not used for this scoping review.

## Results

### Selection of sources of evidence

**Figure 1:**
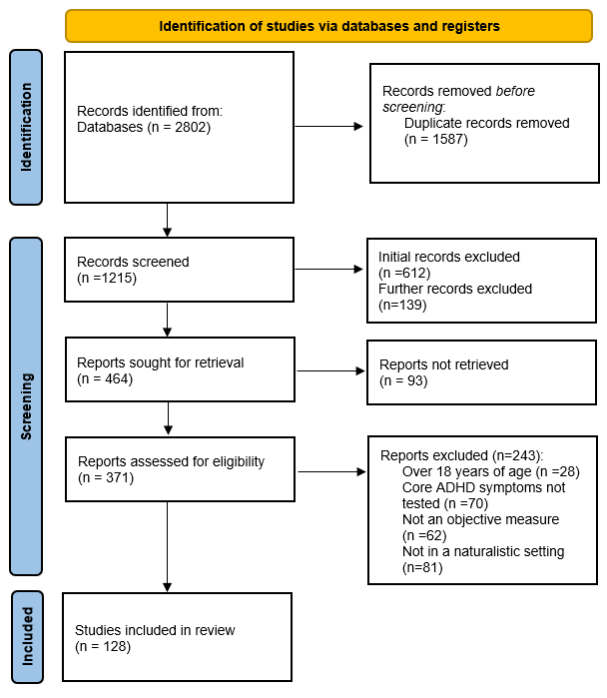
Preferred Reporting Items for Systematic Reviews and Meta-Analyses flow diagram of screened and included studies. ADHD, attention-deficit/hyperactivity disorder.

The screening process is shown in figure 1. The original search yielded 2802 potentially relevant citations. Before screening, 1587 duplicates were removed. After deduplication, 1215 citations proceeded to the screening stage. A total of 612 records were initially excluded at title and abstract screening. Following an alteration to the original protocol, a second stage of title and abstract screening was undertaken to exclude papers that described sleep and ADHD using objective measures, unless papers were reporting on the use of the objective measures to quantify core ADHD symptoms. This resulted in the exclusion of a further 139 papers. A total of 454 papers potentially met the eligibility criteria based on title and abstract, and the corresponding full-text papers were procured for review. Ninety-three papers were unobtainable and were not screened at full-text stage, 361 papers were screened at the full-text stage. In total, 128 papers were eligible for inclusion.

### Characteristics of sources of evidence

A total of 112 of the included papers described primary research and 16 were reviews, 2 of which were systematic reviews. Thirty-nine of the included studies involved understanding, assessing or improving non-pharmacological interventions, 19 involved investigating demographic characteristics in relation to ADHD and associated behaviours or outcomes, 16 tested the psychometric properties of objective measures, 11 involved adapting or evaluating current diagnostic criteria, 10 involved exploring, identifying or evaluating functional impairments associated with ADHD, 9 investigated the efficacy, impact or pharmacological aspects of treatments, 8 involved interventions providing support for activity levels, 6 tested the psychometric properties of a subjective measure, 6 involved the development or revision of subjective measures, 5 employed multiple methods, techniques or modalities to assess or diagnose ADHD and one tested a CPT.

Of the included papers, 123 reported country of setting. Most (n=87) were undertaken in the USA, 11 in the UK; 3 each in Canada, Japan, The Netherlands and Switzerland; 2 each in Australia, Italy, South Korea and Spain; and 1 each in Belgium, China, Germany, New Zealand and Taiwan. This is summarised in table 1.

**Table 1:** Included papers participant characteristics

Characteristics	Frequency of included studies (n)	Percentage (%)
**Publicationyear (n=126)**		
1990	3	2.4
1991–2000	21	16.7
2001–2010	58	46.0
2011–2020	39	31.0
2023	5	4.0
**Country oforigin (n=123)**		
USA	87	70.7
UK	11	8.9
Canada	3	2.4
Japan	3	2.4
The Netherlands	3	2.4
Switzerland	3	2.4
Australia	2	1.6
Italy	2	1.6
South Korea	2	1.6
Spain	2	1.6
Belgium	1	0.8
China	1	0.8
Germany	1	0.8
New Zealand	1	0.8
Taiwan	1	0.8
Total no. of participants		
**Recruited (n=65)**		
1–50	23	
51–100	18	
101–200	9	
201–500	7	
501–1000	5	
>1001	3	
**Gender (% male)**		
41–50	8	9.3
51–60	10	11.6
61–70	19	22.1
71–80	17	19.8
81–90	9	10.5
91–100	23	26.7

### What populations have been included?

Eighty nine (69.5%) of the 128 papers had participants with a diagnosis of ADHD. Of those 89 papers, 49 included research diagnoses (55%), 34 included clinical diagnoses (38%), and 4 included both research and clinical diagnoses (4.5%).

#### Primary studies

Of the 112 primary papers included in the review, 96 reported the number of participants. The number of participants ranged from 1 to 1378, with a mean of 118.6 (SD=247.6, median=44, IQR=81.5). The mean age was 8.6 (SD=2.0), and ages ranged from 3 to 18 years old. Eighty-nine of the 103 primary studies either explicitly stated or provided the information (eg, number of male participants) to calculate the percentage of male participants within the study. The mean percentage of male participants across the primary papers was 75.9% (SD=16.83), ranging from 46.4% to 100%.

Forty four of the 103 primary studies described the ethnicity or nationality of the participants; of these, 41 papers explicitly stated the percentage of participants for each ethnicity or nationality group. Nine different ethnicities were included. The most common ethnicity of participants was white/Caucasian (mean=65%, range=14.6%–100%, median=75% and IQR=42.6%) with 32 references to the percentage of the population who were white/Caucasian and three stating their population was ‘predominantly’ or ‘mostly’ white. The other ethnicities mentioned included black/African American (n included studies=26, range reported in included studies=1%–100%, median=12% and IQR=9.4%), Hispanic/Latino (n=18, range=11.6%–100%, median=11%, IQR=25.1%), Asian (n=5, range=1.9%–21%), Native American/American Indian/Inuit or Aleutian (n=4, range=1%–2%), mixed ethnicity/bi-racial/multiracial (n=7, range=3.7%–21%), Asian Pacific Islander (n=4, range=1%–14%), ‘Ethnic Minority’ (n=1), and other (n=6, range=1.16%–21%).

Rather than ethnicity, some studies reported nationality. Nine different named nationalities were included within the primary papers. This included Portugal (n=1), China (n=1), West Indies (n=1), Japan (n=2), Taiwan (n=1), Former Yugoslavia (n=1), ‘the rest of Europe’ (n=1), African (n=1) and ‘the rest of the world’ (n=1). Further details on participant characteristics can be found in table 1.

#### Reviews

Of the 16 included reviews, three reported the number of participants. The number of participants ranged from 228 to 18 074. Participant ages ranged from 5 to 13 years old. The mean percentage of male participants across the primary papers was 76% (SD=17%), ranging from 46.4%–100%.

### What types of existing objective measures of ADHD in CYP can be applied in naturalistic settings?

The objective measures identified fall into two categories: direct behavioural observations and acceleration-sensitive measures. Of the 83 individual objective measures, 64 (77%) were observational measures and 19 (23%) were acceleration-sensitive measures.

#### Direct behavioural observations (number of included papers=64)

Direct behavioural observations of children’s classroom behaviour were the most common assessment methods used in the included studies (table 2). From the 64 observational measures identified, 22 were unnamed. Our included studies’ direct behavioural observations often used frequency, or event, recording to compare the frequency of behaviours in comparison to control children.^42^ Many used time sampling, which refers to whether a behaviour does or does not occur during an interval within an observation period. This can either be during the whole predefined interval (whole interval), at any point within the interval (partial interval) or at a fixed moment of time (momentary time sampling).^24 43^

**Table 2:** Reliability reported in included direct behavioural observation studies (n=64)

Objective measure	First author	Year	Percentage agreement	Kappa	Other interobserver value (name)	Test–retest
The Behaviour Observation System for Schools (BOSS)	Stevens^47^	1998	81	–	–	–
	Volpe^81^	2003	88–99	–	–	–
	DuPaul *et al*^82^	2004	91.5–99.27	–	–	–
	Hoff *et al*^83^	2005	94.03	–	–	–
	Vile Junod and DuPaul^44^	2006	–	0.93 to 0.98	–	–
	Hosterman *et al*^84^	2008	–	0.89 to 0.96	–	–
	Pfiffner *et al*^45^	2013	–	0.86	–	–
	Steiner *et al*^85^	2014	86	–	–	–
	Steiner *et al*^86^	2014	–	0.86	–	–
	Steiner *et al*^46^	2014	–	0.89	–	–
	Slattery *et al*^87^	2016	80.5–100	–	–	–
	Simpson^88^	2016	97.22	–	–	–
	Kennerley *et al*^48^	2018	90%, 84%	–	–	–
	Minder *et al*^24^	2018	94%	0.67		
	Jiang *et al*^89^	2019	–	0.83		
	Meza *et al*^90^	2020	72–83	–	–	–
	Ramer *et al*^91^	2020	80	–	–	–
	Minder *et al*^24^	2018	–	0.77– 0.98	–	–
	Staff *et al*^30^	2021	–	0.83	–	–
Classroom Observation Code (COC)	Gadow *et al*^92^	1991	–	0.76–1.0	–	–
	Abikoff *et al*^93^	2002	–	–	0.80–1.00 (Phi)	–
	Miller *et al*^94^	2004	86	0.72–1.0	–	–
	Veenman *et al*^50^	2017	–	–	0.77 (α)	–
	Minder *et al*^24^	2018	–	–	0.77–0.94 (Phi), 0.80–1 (α)	–
	Staff *et al*^30^	2021	–	–	0.77 to >0.87 (Phi)	–
	Abikoff *et al*^95^	2004	–	–	0.83–0.92 (Phi)	–
	Stevenson *et al*^52^	2010	–	–	0.87 (Phi)	–
	Volpe *et al*^96^	2009	–	–	0.71 to 0.97 (α)	0.25–0.77 (α)
	McConaughy *et al*^97^	2010	–	–	0.71–0.80 (α)	–
	Johnson *et al*^54^	2020	87–91	–	–	–
	Minder *et al*^24^	2018	–	–	0.57–1 (r)	r = 0.25–0.77
	Staff *et al*^30^	2021	72–80	–	–	–
	Skansgaard and Burns^98^	1998	97	–	–	–
The ADHD Behaviour Coding System	DuPaul and Barkley^99^	1992	94.8	–	–	–
The Teacher Pupil Interaction Scale (TPIS)	Dunson Ill *et al*^100^	1994	91	–	–	–
The Code for Observing Social Activity (COSA)	Nolan and Gadow^101^	1994	–	–	–	0.07–0.24 (ICC)
	Gadow *et al*^92^	1991	95	–	–	–
On-/Off-Task Behaviour Observation Form	Thompson^102^	1994	69–88	–	–	0.12–0.57 (ICC)
Adapted from the BOSS	Hoff *et al*^83^	2005	94.03	–	–	–
Functional Observation of Classrooms and Learners (FOCAL Point)	Kenney *et al*^64^	2004	98	0.53	–	0.53
Adapted from Hinshaw, Han, Erhardt, and Huber (1992) and Hinshaw (1993)	Wood *et al*^103^	2002	0.74–0.93	–	–	–
Adaptation of Barkley’s ‘restricted academic situation coding sheet’	Austin^104^	2003	93.20	–	–	–
An adaptation of the Restricted Academic Task	Hale *et al*^105^	2005	≥0.90	–	–	–
Responses to Interpersonal and Physically Provoking Situations (RIPPS)	Carroll *et al*^106^	2006	78.19	–	–	–
	Minder *et al*^24^	2018	80%	–	–	–
Barkley’s ADHD Behaviour Coding System	Luitjohan^107^	2005	–	–	–	–
The SPA Behaviour Observation Form (SPA-BOF)	Simonsen and Bullis^108^	2007	86.1–87	–	–	–
The Abikoff Structured School Observation Code	Riley *et al*^109^	2008	–	0.74–0.83	–	–
Adapted the Social Behaviour Observation System of the Early Screening Procedure	McGoey *et al*^110^	2010	96.10	–	–	–
The Scope Classroom Observation Checklist (SCOC)	Scope *et al*^62^	2007	98.9	–	–	–
Classroom Behaviour Record (CBR)	Milich and Landau^111^	1988	86–100	–	–	–
Classroom Observations of Conduct and Attention Deficit Disorders (COCADD)	Atkins *et al*^112^	1989	–	0.86–0.9	–	–
ADHD School Observation Code (ADHD-SOC)	Minder *et al*^24^	2018	–	0.57–0.84	–	–
On-task percentage	Staff *et al*^30^	2021	–	0.74	–	–
ADHD behaviour code	Staff *et al*^30^	2021	–	0.56	–	–
Ghent University Classroom Coding Inventory (GUCCI)	Staff *et al*^30^	2021	–	0.77–0.99	–	–
No name 1	Mcnamara and Jolly^113^	1990	95.70	–	–	–
No name 2	Bloomquist *et al*^114^	1991	95	–	–	–
No name 3	DuPaul^115^	1991	90	0.74	–	–
No name 4	Grossman^116^	1991	81–92	–	–	–
No name 5	DuPaul and Barkley^99^	1992	90	0.74	–	–
No name 6	Charlebois *et al*^117^	1993	–	–	0.74 (α)	–
No name 7	Broussard and Northup^118^	1995	80–100	–	–	–
No name 8	Rapport and Denney^119^	1997	92.4	0.84	–	–
No name 11	Murray^120^	2002	75–100	–	–	–
No name 12	Ardoin and Martens^121^	2004	97	–	–	–
No name 13	Mclaughlin *et al*^122^	2003	95	–	–	–
No name 14	DuPaul *et al*^123^	2006	85.18–99.98	–	–	–
No name 15	Hoerger and Mace^124^	2006	96	–	–	–
No name 16	Symons *et al*^125^	2007	>85	–	–	–
No name 17	Rapport *et al*^126^	2009	92.40	0.84	–	–
No name 18	Wheeler *et al*^34^	2009	95–100	–	–	–
No name 19	Fedewa and Erwin^127^	2011	93	–	–	–
No name 20	Schafer *et al*^128^	2013	98	–	–	–
No name 21	Aspiranti and Hulac^63^	2022	98.5	–	–	–

.ADHDattention-deficit/hyperactivity disorderICC, intraclass correlationPhi, phi coefficient; r, Pearson’s r; α, Cronbach’s alpha

The most frequently used measure was the Behaviour Observation System for Schools (BOSS) (n=24). The BOSS is a whole-interval time sampling measure which is split into 15-s intervals. The total observation period was predominantly 15 min across the studies,^44^,^46^ however, some took 10^47^ or 30 min.^48^ There are five behaviour codes used across the studies which used the BOSS: Active Engaged Time (AET), Passive Engaged Time (PET), Off Task Motor (OFT-M), Off Task Verbal (OFT-V), and Off Task Passive (OFT-P).

The next most frequently identified was the Classroom Observation Code (COC) (n=16, 26%). Similar to the BOSS, the COC is a whole-interval time sampling measure which is split into 15-s intervals. Again, the total length differed between studies (eg, 30 min,^49^ across two blocks of 8 min^50^ and 2/3 min^51^). The COC assesses the occurrence of 12 mutually exclusive behaviour categories including Interference, Off-Task Behaviour, Non-Compliance, Gross Motor-Standing, Gross Motor-Vigorous, Out-of-Chair Behaviour, Physical Aggression, Threat or Verbal Aggression Directed at Another Child, Threat or Verbal Aggression Directed at the Teacher, Solicitation of Teacher, and Absence of Behaviour.^52^

The third most frequently identified was the Direct Observation Form (DOF) (n=12). The DOF is a 10-min observation which can occur in a group, classroom or playtime setting.^53^ As well as a narrative description (that would not meet criteria for being an objective measure), the observer rates whether the child is on-task or off-task during the last 5 s of each 1 min-interval.^54^ The DOF On-task score is the total number of 1-min intervals when the child was rated as on-task, averaged across multiple 10-min observations.^52^

A summary of each observational measure is provided in the online supplemental table 1.

#### Acceleration-sensitive measures (n=19)

Acceleration-sensitive measures measure activity levels or movement of an individual.^37^ The type of acceleration-sensitive measure common in our review was actigraph (n=19). Actigraphs are small, sensor-based devices that record motor activity.^55^ They are usually worn on the wrist (n=16), waist (n=5), hip (n=4) or ankle (n=3) (some were worn in multiple places). Actigraphs calculate an individual’s activity level per unit of time,^56^ and can record continuously over hours or days.^57 58^ Due to this, they are useful for measuring habitual activity in everyday life.^59^ However, it has been suggested that actigraphy simplifies complex motor data into the number of times that part of the body accelerates above a pre-set threshold.^56^

The most frequently mentioned acceleration-sensitive measure was the motionlogger actigraph (including, mini and micro mini) (n=8), which is a device that was worn continuously on the non-dominant wrist^53 56 60^ or as a belt.^61^ The next most frequently mentioned acceleration-sensitive measures were the CSA actigraph (n=2), the actigraph LIGNEX 1 (n=2), GT1 M actigraph (n=2), Runscribe inertial sensors (n=2) and actigraph GT3x device (n=2).

A summary of each observational measure is provided in online supplemental table 2.

#### What is the reliability and validity of the objective measures?

##### Reliability

Over half of the papers mentioned the reliability of the objective measure (n=76). Reliability was predominantly expressed as inter-rater reliability (n=80), with some using test–retest (n=5). Inter-rater reliability was mainly presented as a percentage agreement (n=48), kappa statistic (n=21), phi coefficient (n=5) and Cronbach’s alpha (n=6). Further reliability details are reported in table 2. The measures found to be most reliable between raters were an unnamed measure (% agreement=1), the Scope Classroom Observation Checklist (SCOC) (% agreement=0.989),^62^ and the Behavioural Observation of Students in Schools (BOSS) (% agreement=0.985).^63^ The lowest reliability measure was a phi coefficient of 0.53 for the Functional Observation of Classrooms and Learners (FOCAL Point).^64^

##### Validity

Aspects of validity were mentioned in 11 papers but often inconsistently. Where papers only used correlation coefficients to quantify agreement, these statistics are not reported here. Group comparisons were reported when comparing ADHD groups to control groups. Summaries of validity findings in these papers are presented in table 3.

**Table 3:** Validity reported in included studies

Reference	Objective measure	Summary of validity
Skansgaard 1998^98^	DOF	The control group consistently showed the lowest symptoms and highest on-task behaviour (M=0.69, SD=0.76 for inattention; M=1.58, SD=1.67 for hyperactivity/impulsivity; M=0.77, SD=1.34 for OCD/ODD; M=0.27, SD=0.39 for SCT; M=9.52, SD=0.61 for on-task behaviour). The ADHD-CT group showed the highest levels of hyperactivity/impulsivity (M=8.25, SD=3.66) and OCD/ODD symptoms (M=4.13, SD=1.55). The ADHD-IT group exhibited higher inattention (M=6.58, SD=3.2) and SCT symptoms (M=1.04, SD=0.97) compared with the control group, but lower hyperactivity/impulsivity (M=4.00, SD=3.57) and OCD/ODD symptoms (M=1.21, SD=1.44) compared with the ADHD-CT group.
Tsujii 2009^56^	Mini-Motionlogger	Group differences in activity levels between boys with ADHD and control group. No significant differences were found across average activity levels in continuous (ADHD: M=7.72, SD=2.16; Controls: M=8.22, SD=1.56), in-seat (ADHD: M=214.54, SD=14.42; Controls: M=210.67, SD=26.76) or recess periods (ADHD: M=244.94, SD=12.29; Controls: M=241.32, SD=15.16).
Abikoff 2002^93^	COC	The observations effectively differentiated between ADHD and control group for both girls and boys (p<0.001), except for one observed behaviour (Solicitation=p<0.003). For boys, there were significant differences between ADHD and comparison (p<0.001), except for one observed behaviour (Solicitation=p<0.003). For girls, there were significant differences between ADHD and comparison, but not across all categories.
DuPaul 2004^82^	BOSS	Group differences in achievement and predictor variables between ADHD group and controls. For reading, significant differences are observed in Passive Engaged Time (PET) (0.001), Off-Task (OFT) (<0.001), and Non-Compliant (NonC) (0.03) scores. For maths, significant differences are observed in PET (<0.001), OFT (<0.001) and NonC (0.02) scores. Non-significant differences were found in Active Engaged Time (AET) for both reading (0.40) and math (0.12).
McGrath 2004^129^	Restricted Academic Situation (RAS)	The Restricted Academic Situation (RAS) did not reveal significant differences between groups when examining total scores or any subscales independently.
	ActiTrac activity monitor	The activity monitor demonstrated significant group differences. Significant differences were observed in the sum of activity across 3 days, F_2,87_ =4.23, p<0.025. Post hoc analysis further highlighted that the ADHD-Both groups exhibited significantly higher activity levels compared with the control group (Tukey’s HSD=17.42, p<0.05). The ADHD-CT group did not differ significantly from either the ADHD-Both or control groups in terms of activity levels.
Vile Junod 2006^45^	BOSS	The BOSS accurately classified group membership (ADHD vs controls) with high accuracy: 92.2% accuracy when combined with academic scores and SES, and 70.6% accuracy with just observational variables.
Kam 2010^59^	LIG Nex1 Co., Ltd., Yongin, Korea	Two decision-trees, a supervised learning algorithm, were constructed utilising accelerometer data. Model A (class and playtime), achieved Acc of 99.3%, PPV of 1.00, NPV of 0.992, Sens of 0.803, Spec of 0.909, LR of 1.00 and AUC of 1.00. Model B (class only), achieved Acc of 98.59%, PPV of 1.00, NPV of 0.985, Sens of 0.671, Spec of 0.832, LR of 1.00 and AUC of 1.00.
Faedda 2016^79^	Mini-Motion logger	Participants with ADHD exhibited distinct patterns in several key measures compared with controls. Notably, ADHD showed significant deviations in activity parameters such as ‘Diurnal Skew’ (p<0.02), ‘% Very Low Activity’ (p<0.04) and ‘% Low Activity’ (p<0.05).
Muñoz-Organero 2018^58^	Runscribe inertial sensors	The wrist accelerometer showed an Acc of 0.9375, Sens of 1.00 and Spec of 0.9091. The ankle accelerometer exhibited an Acc of 0.9375, with Sens of 0.80 and Spec of 1.00.
Amado-Caballero 2020^130^	ActiGraph GT3x device	Diagnosis is based on the analysis of 24 hour-long activity records using CNN, a type of deep learning algorithm, to classify activity windows. For a window size of 1800 s, CNN 2D-3 achieved an Acc of 0.9643 (SD=0.0302), Sens of 0.9429 (SD=0.0514), Spec of 0.9857 (SD=0.023), AUC of 0.9980 (SD=0.029), PPV of 0.9854 (SD=0.0235), NPV of 0.9474 (SD=0.0483), LR+ of 18 and LR− of 0.0580 (SD=0.055). CNN 1D-3 in the same window size showed Acc of 0.5571 (SD=0.0999), Sens of 0.3762 (SD=0.2158), Spec of 0.7381 (SD=0.1128), AUC of 0.5755 (SD=0.1359), PPV of 0.5727 (SD=0.1666), NPV of 0.5532 (SD=0.0825), LR+ of 0.4286 and LR− of 0.8451 (SD=0.2811).

.Acc, accuracy; AUC, area under the curveCNNconvolutional neural networksLR, likelihood ratio; NPV, negative predictive value; OCD/ODDObsessive Compulsive Disorder/Obsessive Defiant DisorderPPV, positive predictive value; SCTSluggish Cognitive TempoSens, sensitivity; Spec, specificity

### How were the objective measures implemented?

#### Implementation

Of the 128 primary and review papers, 16 identified the format of the measure. This does not include papers where the observational measure records what it does (eg, accelerometers). Twelve of the 16 measures were recorded electronically and four used pencil and paper, as seen in online supplemental tables 3 and 4.

Forty seven of the 128 papers mentioned who recorded the objective measure, as seen in online supplemental tables 3 and 4. Of the 103 primary papers, 47 mentioned training being given or stated the observers were ‘trained’. Five of those papers that mentioned training, also mentioned the length of time the training took. The length of training time ranged from 3 hours to 1 year. More detail is shown in online supplemental tables 3 and 4. Of the 103 primary papers, 85 mentioned how long the measure took to complete. The observational measures ranged from 5 min to 90 min, with many being repeated multiple times. The acceleration-sensitive measures ranged from 3 hours to >7 days.

## Discussion

Given the increasing interest in objective measures, this review aimed to understand the range and types of objective measures of ADHD in the research literature that are relevant to CYP and that could be applied in naturalistic settings.

The review found 83 objective measures; systematic behavioural observations and acceleration-sensitive measures made up most of the objective measures used in naturalistic settings, with the same large-scale commercialised tests (eg, the BOSS) being predominantly used across papers. Like Minder *et al*,^24^ we found the BOSS to be one of the most commonly used objective measures of ADHD. However, alterations to measures, like the BOSS test, lack consistent documentation. We found considerably less included studies usedacceleration-sensitive measures, with just over 30% of the papers reporting on them. This reflects Hall *et al*, who similarly found acceleration-sensitive tests to be less commonly used than observations.^29^ This is perhaps unsurprising as there is no mandatory requirement for psychological or neuropsychological tests in the diagnostic process, highlighting a potential gap in assessment practices.

The included studies primarily aimed to refine assessment methods and assess the efficacy of interventions, particularly school-based cognitive behavioural therapy programmes, often integrating objective measures alongside conventional assessment methods. This is reflective of best-practice recommendations in literature, such as those made by Emser *et al*,^28^ who highlight the added value of using an objective measure in ADHD assessment, as well as being reflective of clinical guidance. Despite this, these are less used in clinical practice.^29 35^ This could be for numerous reasons, one being that the psychometric properties of one objective measure are reported to vary widely across studies, as seen in this review. Clinician and researcher had confidence that objective measures that capture change or symptoms robustly may be impacted by this, leading to objective measures being used as an adjunct rather than a primary outcome or assessment method. This review shows that there are, however, objective measures that are psychometrically sound, such as the BOSS; there remains a gap between research findings and real-world application, highlighting the need for further bridging of this divide to improve clinical outcomes. Moreover, the majority of the included papers were from the USA, which was considerably higher than the next most common setting (the UK (9%)). This was unsurprising as psychological papers remain largely American.^65^ While only making up 4.25% of the total world population, the USA dominates psychological research.^66 67^ This influence is noteworthy, particularly regarding variations in how ADHD is understood across cultures. Psychological research, often reliant on White, Educated, Industrialised, Rich and Democratic (WEIRD) samples, risks limited generalisability.^68^ Objective measures developed and assessed solely in Western contexts may not be universally applicable and could even be harmful.^69 70^ Despite increased publications from non-Western countries since 2014, the Global South remains under-represented in top social science outputs.^71^ In our study, only 0.8% of papers were from the Global South, with China being the sole representative. Further cross-cultural validation of objective measures is essential for robust generalisation.

Another area warranting further scrutiny is the reporting of psychometric properties of measures. In our review, over half of the papers reported reliability, and only 11 reported validity. Previous studies have identified a systematic lack of reporting of psychometric properties across the fields of education, health and psychology.^72 73^ Echoing Barry *et al*,^72^ the most reported psychometric property in this review was inter-rater reliability. Similar to wider literature,^74^,^76^ validity was significantly under reported, with only 11 included papers reporting validity. It could be suggested that researchers assume that a given measure will be sufficient either based on previous studies or the common use of the measure,^77^ however, as many also lack adequate reporting this could create further issues if relying on these measures. Reassessing reliability and validity statistics, even if reported in previous studies, to guide instrument selection is crucial for establishing psychometric evidence in the new sample.^78^ Following the synthesis of our findings, a psychometric reporting guideline has been published by Johansson *et al*.^79^ They provide both a minimal checklist, for what readers should scrutinise in a psychometric paper, and a comprehensive set of checklist items for studies reporting psychometric properties. They go on to suggest that the minimal checklist could act as a foundation for determining whether a measure is valid and reliable. Future research studies should focus on clearly stating how objective measures are used, the processes behind using and recording objective measures, and routinely report psychometric properties of the measures in the study sample as well as citing those from prior papers. Using a reporting guideline will ensure more consistency and a higher standard across psychometric reporting. Encouragingly, recent papers begin to address these issues. For example, Basic *et al*^80^ explored the use of motion sensors for detecting ADHD and found high accuracy with advanced computational methods. However, they emphasise a need for further validation and integration with other methods.

Based on this review, clinicians integrating objective measures into assessment, particularly in naturalistic settings like schools, should prioritise measures with strong psychometric evidence. Further research is needed to validate objective measures across diverse populations, including non-Western cultures, to improve generalisability. Methodological transparency, including clear definitions of terms and detailed descriptions of methodologies, is crucial for advancing ADHD research.

Our scoping review was the first to identify objective measures in naturalistic settings, specifically for CYP. One strength was that this scoping review followed the PRISMA-ScR guidelines to ensure a high standard of methodological and reporting quality. We further endeavoured to report changes to criteria from the protocol, in order to be transparent with our identification process. An arguable weakness is the arbitrariness in definition of objective and naturalistic. The term objective can be understood differently, and our criteria within this paper may limit the scope of our findings. Naturalistic was defined as something which does not take a child out of their normal routine, however the definition of a ‘normal routine’ could be contested. Hence, this generalisation may have missed some papers. This is especially true where children with special educational needs, including children with ADHD, may be taken out of the classroom in smaller groups or work 1-to-1.

This study identified multiple objective measures of ADHD, which could be used to assess symptoms of ADHD in naturalistic settings (eg, school). However, the searches were conducted in 2022, and publication in 2024 therefore means there may have been further relevant studies published that are not captured within our findings. Throughout this review it has been evident that the psychometric properties and implementation of objective measures has been overlooked in ADHD research in naturalistic settings. Further adaptations and testing would be needed to see if objective measures were valid for a range of neurodevelopmental disorders or to add value to diagnostic decisions. Further testing is needed in a variety of cultures and countries to be able to generalise our findings, particularly in the Global South.

## Conclusion

In conclusion this review identified a range of measures to objectively measure traits of ADHD. It highlighted a need for improved psychometric reporting, as well as transparency of how measures were implemented. Regarding specific measures, the BOSS is the most used in research and has the most recordings of its psychometric properties. However, there are some promising measures that require further research (eg, the SCOC).

## supplementary material

10.1136/bmjopen-2023-080306 online supplemental file 1

## Data Availability

All data relevant to the study are included in the article or uploaded as supplementary information.

## References

[1] Polanczyk G, de Lima MS, Horta BL (2007). The worldwide prevalence of ADHD: a systematic review and metaregression analysis. Am J Psychiatry.

[2] Agnew-Blais J, Michelini G (2023). Taking stock of the present and looking to the future of ADHD research: a commentary on Sonuga-Barke etal. (2023). J Child Psychol Psychiatry.

[3] May F, Ford T, Janssens A (2021). Attainment, attendance, and school difficulties in UK primary schoolchildren with probable ADHD. Br J Educ Psychol.

[4] Sasser T, Schoenfelder EN, Stein MA (2017). Targeting functional impairments in the treatment of children and adolescents with ADHD. CNS Drugs.

[5] Chen M-H, Hsu J-W, Huang K-L (2018). Sexually transmitted infection among adolescents and young adults with attention-deficit/hyperactivity disorder: a nationwide longitudinal study. J Am Acad Child Adolesc Psychiatry.

[6] Howard AL, Kennedy TM, Mitchell JT (2020). Early substance use in the pathway from childhood attention-deficit/hyperactivity disorder (ADHD) to young adult substance use: evidence of statistical mediation and substance specificity. Psychol Addict Behav.

[7] Lidestam B, Selander H, Vaa T (2021). The effect of attention-deficit/hyperactivity disorder (ADHD) on driving behavior and risk perception. Traffic Inj Prev.

[8] Hinshaw SP (2018). Annual review of clinical psychology Attention Deficit Hyperactivity Disorder (ADHD): controversy, developmental mechanisms, and multiple levels of analysis.

[9] American Psychiatric Association (2013). DSM-5 handbook of differential diagnosis.

[10] Castellanos FX, Sonuga-Barke EJS, Milham MP (2006). Characterizing cognition in ADHD: beyond executive dysfunction. *Trends Cogn Sci*.

[11] Hellwig L (2017). Observation of ADHD symptoms: prospects for a behavior-based, objective and context-dependent assessment.

[12] NICE (2018). Attention deficit hyperactivity disorder: diagnosis and management NICE guideline.

[13] Coghill DR, Werner-Kiechle T, Farahbakhshian S (2021). Functional impairment outcomes in clinical trials of different ADHD medications: post hoc responder analyses and baseline subgroup analyses. Eur Child Adolesc Psychiatry.

[14] Fuchs T (2010). Subjectivity and intersubjectivity in psychiatric diagnosis. Psychopathology.

[15] Jungersen CM, Lonigan CJ (2021). Do parent and teacher ratings of ADHD reflect the same constructs? A measurement invariance analysis.

[16] Takeda T, Nissley-Tsiopinis J, Nanda S (2020). Factors associated with discrepancy in parent-teacher reporting of symptoms of ADHD in a large clinic-referred sample of children. J Atten Disord.

[17] Bhugra D, Easter A, Mallaris Y (2011). Clinical decision making in psychiatry by psychiatrists. Acta Psychiatr Scand.

[18] Chung J, Tchaconas A, Meryash D (2016). Treatment of attention-deficit/hyperactivity disorder in preschool-age children: child and adolescent psychiatrists’ adherence to clinical practice guidelines. J Child Adolesc Psychopharmacol.

[19] Dekkers TJ, Groenman AP, Wessels L (2022). Which factors determine clinicians’ policy and attitudes towards medication and parent training for children with attention-deficit/hyperactivity disorder?. *Eur Child Adolesc Psychiatry*.

[20] Kovshoff H, Williams S, Vrijens M (2012). The decisions regarding ADHD management (DRAMa) study: uncertainties and complexities in assessment, diagnosis and treatment, from the clinician’s point of view. Eur Child Adolesc Psychiatry.

[21] Nelson JM, Whipple B, Lindstrom W (2019). How Is ADHD assessed and documented? Examination of psychological reports submitted to determine eligibility for postsecondary disability. J Atten Disord.

[22] Hult N, Kadesjö J, Kadesjö B (2018). ADHD and the QbTest: diagnostic validity of QbTest. J Atten Disord.

[23] Escolano-Pérez E, Herrero-Nivela ML, Blanco-Villaseñor A (2017). Systematic observation: relevance of this approach in preschool executive function assessment and association with later academic skills. Front Psychol.

[24] Minder F, Zuberer A, Brandeis D (2018). A review of the clinical utility of systematic behavioral observations in Attention Deficit Hyperactivity Disorder (ADHD). Child Psychiatry Hum Dev.

[25] Gawrilow C, KÃ¼hnhausen J, Schmid J (2014). Hyperactivity and motoric activity in ADHD: characterization, assessment, and intervention. Front Psychiatry.

[26] Griffiths KR, Quintana DS, Hermens DF (2017). Sustained attention and heart rate variability in children and adolescents with ADHD. Biol Psychol.

[27] Cortese S, Kelly C, Chabernaud C (2012). Reviews and overviews mechanisms of psychiatric illness toward systems neuroscience of ADHD: a meta-analysis of 55 fMRI studies. Am J Psychiatry.

[28] Emser TS, Johnston BA, Steele JD (2018). Assessing ADHD symptoms in children and adults: evaluating the role of objective measures. *Behav Brain Funct*.

[29] Hall CL, Valentine AZ, Groom MJ (2016). The clinical utility of the continuous performance test and objective measures of activity for diagnosing and monitoring ADHD in children: a systematic review. Eur Child Adolesc Psychiatry.

[30] Staff AI, Oosterlaan J, Oord S Psychometric properties of an observation instrument to assess ADHD symptoms in the classroom using a continuous sampling approach.

[31] Wang XQ, Albitos PJ, Hao YF (2022). A review of objective assessments for hyperactivity in attention deficit hyperactivity disorder. J Neurosci Methods.

[32] Wang MT, Degol JL, Amemiya J (2020). Classroom climate and children’s academic and psychological wellbeing: a systematic review and meta-analysis.

[33] Rommelse N, Bunte T, Matthys W, Anderson E, Buitelaar J, Wakschlag L. (2015). Contextual variability of ADHD symptoms: embracement not erasement of a key moderating factor.

[34] Wheeler L, Pumfrey P, Wakefield P (2009). Variability of ADHD symptoms across primary school contexts: an in‐depth case study. Emot Behav Diffic.

[35] Gualtieri CT, Johnson LG (2005). ADHD: is objective diagnosis possible?. Psychiatry (Edgmont).

[36] Sonuga‐Barke EJS, Fearon RMP (2019). Commentary: ‘Ready or not here I come’: developmental immaturity as a driver of impairment and referral in young‐for‐school‐gradeADHDchildren. A reformulation inspired by Whitely etal. (2019). Child Psych Psychiatry.

[37] Loh HW, Ooi CP, Barua PD (2022). Automated detection of ADHD: current trends and future perspective. Comput Biol Med.

[38] Munn Z, Peters MDJ, Stern C (2018). Systematic review or scoping review? Guidance for authors when choosing between a systematic or scoping review approach. BMC Med Res Methodol.

[39] Tricco AC, Lillie E, Zarin W (2016). A scoping review on the conduct and reporting of scoping reviews. *BMC Med Res Methodol*.

[40] Tricco AC, Lillie E, Zarin W (2018). PRISMA Extension for Scoping Reviews (PRISMA-ScR): checklist and explanation. Ann Intern Med.

[41] Kelman CR, Thompson Coon J, Ukoumunne OC Objective measures of core ADHD symptoms in children and young people in naturalistic settings: a scoping review protocol.

[42] Smith M (2017). Hyperactive around the world? The history of ADHD in global perspective. Soc Hist Med.

[43] Dowdy E, Twyford J, Sharkey JD (2013). Methods of assessing behavior: observations and rating scales.

[44] Junod REV, DuPaul GJ (2005). The impact of ADHD on elementary school functioning: a comparison across ethnic groups. ADHD Rep.

[45] Pfiffner LJ, Villodas M, Kaiser N (2013). Educational outcomes of a collaborative school-home behavioral intervention for ADHD. Sch Psychol Q.

[46] Steiner NJ, Frenette EC, Rene KM (2014). Neurofeedback and cognitive attention training for children with attention-deficit hyperactivity disorder in schools. J Dev Behav Pediatr.

[47] Stevens ML (1998). Effects of classwide peer tutoring on the classroom behavior and academic performance of students with ADHD.

[48] Kennerley S, Jaquiery B, Hatch B (2018). Informant discrepancies in the assessment of attention-deficit/hyperactivity disorder. J Psychoeduc Assess.

[49] Tiah TM (2013). Predictability of ADHD behavioral symptoms: a follow-up examination in at-risk preschool children.

[50] Veenman B, Luman M, Oosterlaan J (2017). Further insight into the effectiveness of a behavioral teacher program targeting ADHD symptoms using actigraphy, classroom observations and peer ratings. Front Psychol.

[51] Miller TW, Nigg JT, Miller RL (2009). Attention deficit hyperactivity disorder in African American children: what can be concluded from the past ten years?. Clin Psychol Rev.

[52] Stevenson J, Sonuga-Barke E, McCann D (2010). The role of histamine degradation gene polymorphisms in moderating the effects of food additives on children’s ADHD symptoms. Am J Psychiatry.

[53] Schottelkorb AA (2007). Effectiveness of child-centered play therapy and person-centered teacher consultation on ADHD behavioral problems of elementary school children: a single case design. J Dev Behav Pediatr.

[54] Johnson KA, White M, Wong PS (2020). Aspects of attention and inhibitory control are associated with on-task classroom behaviour and behavioural assessments, by both teachers and parents, in children with high and low symptoms of ADHD. Child Neuropsychol.

[55] Corkum P, Panton R, Ironside S Acute impact of immediate release methylphenidate administered three times a day on sleep in children with attention-deficit/hyperactivity disorder. J Pediatr Psychol.

[56] Tsujii N, Okada A, Kaku R (2009). Differentiation between attention‐deficit/hyperactivity disorder and pervasive developmental disorders with hyperactivity on objective activity levels using actigraphs. Psychiatry Clin Neurosci.

[57] Licht CA (2005). Does actigraphy validate DSM-IV criteria and methods for diagnosing pervasive hyperactivity.

[58] Muñoz-Organero M, Powell L, Heller B (2019). Using recurrent neural networks to compare movement patterns in ADHD and normally developing children based on acceleration signals from the wrist and ankle. Sensors (Basel).

[59] Kam HJ, Shin YM, Cho SM (2010). Development of a decision support model for screening attention-deficit hyperactivity disorder with actigraph-based measurements of classroom activity. Appl Clin Inform.

[60] Ogino K, Takahashi H, Nakamura T (2018). Negatively skewed locomotor activity is related to autistic traits and behavioral problems in typically developing children and those with autism spectrum disorders. Front Hum Neurosci.

[61] Faedda GL, Ohashi K, Hernandez M (2016). Actigraph measures discriminate pediatric bipolar disorder from attention-deficit/hyperactivity disorder and typically developing controls. J Child Psychol Psychiatry.

[62] Scope A, Empson J, McHale S (2007). The identification of children with behavioural manifestations of inattention, hyperactivity and impulsivity, in mainstream school: the development of the scope classroom observation checklist. Emot Behav Diffic.

[63] Aspiranti KB, Hulac DM (2022). Using fidget spinners to improve on-task classroom behavior for students with ADHD. Behav Anal Pract.

[64] Kenney M, Ninness C, Rumph R (2004). Paradoxical patterns in the measurement of hyperactivity. Behav Soc Iss.

[65] Rad MS, Martingano AJ, Ginges J (2018). Toward a psychology of *Homo sapiens*: making psychological science more representative of the human population. Proc Natl Acad Sci U S A.

[66] Arnett JJ (2008). The neglected 95%: why American psychology needs to become less American. Am Psychol.

[67] Bajwa N ul H, König CJ (2019). How much is research in the top journals of industrial/organizational psychology dominated by authors from the U.S.?. *Scientometrics*.

[68] Henrich J, Heine SJ, Norenzayan A (2010). The weirdest people in the world?.

[69] Mishu MP, Tindall L, Kerrigan P Cross-culturallyadapted psychological interventions for the treatment of depression and/or anxiety among young people: a scoping review. *PLoS ONE*.

[70] Ngwenya N, Dziva Chikwari C, Seeley J (2023). Are concepts of adolescence from the global north appropriate for Africa? A debate. BMJ Glob Health.

[71] Thalmayer AG, Toscanelli C, Arnett JJ (2021). The neglected 95% revisited: is American psychology becoming less American?. Am Psychol.

[72] Barry AE, Chaney B, Piazza-Gardner AK (2014). Validity and reliability reporting practices in the field of health education and behavior: a review of seven journals. Health Educ Behav.

[73] Ntumi S, Twum Antwi-Agyakwa K A systematic review of reporting of psychometric properties in educational research. *MEDITERR J SOC BEH RES*.

[74] Flake JK, Fried EI (2020). Measurement schmeasurement: questionable measurement practices and how to avoid them. Adv Methods Pract Psychol Sci.

[75] Hussey I, Hughes S (2020). Hidden invalidity among 15 commonly used measures in social and personality psychology. Adv Meth Pract Psychol Sci.

[76] Schimmack U (2021). The validation crisis in psychology. *MP*.

[77] Lilienfeld SO, Strother AN (2020). Psychological measurement and the replication crisis: four sacred cows. Can Psychol Psychol can.

[78] Hagan TL (2014). Measurements in quantitative research: how to select and report on research instruments. Oncol Nurs Forum.

[79] Johansson M, Preuter M, Karlsson S (2023). Valid and reliable? basic and expanded recommendations for psychometric reporting and quality assessment. Open Science Framework.

[80] Basic J, Uusimaa J, Salmi J (2024). Digital Health and Wireless Solutions.

[81] Volpe RJ. (2003). Effects of two academic intervention protocols on the disruptive classroom behavior of children with ADHD.

[82] DuPaul GJ, Volpe RJ, Jitendra AK (2004). Elementary school students with AD/HD: predictors of academic achievement. J Sch Psychol.

[83] Hoff KE, Ervin RA, Friman PC (2005). Refining functional behavioral assessment: analyzing the separate and combined effects of hypothesized controlling variables during ongoing classroom routines. School Psych Rev.

[84] Hosterman SJ, DuPaul GJ, Jitendra AK (2008). Teacher ratings of ADHD symptoms in ethnic minority students: bias or behavioral difference?. Sch Psychol Q.

[85] Steiner NJ, Frenette EC, Rene KM (2014). In-school neurofeedback training for ADHD: sustained improvements from a randomized control trial. Pediatrics.

[86] Steiner NJ, Sheldrick RC, Frenette EC (2014). Classroom behavior of participants with ADHD Compared with Peers: Influence of Teaching Format and Grade Level. J Appl Sch Psychol.

[87] Slattery L, Crosland K, Iovannone R (2016). An evaluation of a self-management intervention to increase on-task behavior with individuals diagnosed with attention-deficit/hyperactivity disorder. J Posit Behav Interv.

[88] Simpson JF (2016). The calming effects of modified lighting.

[89] Jiang Y, Capriotti M, Beaulieu A (2019). Contribution of the behavioral observation of students in schools to ADHD assessment. School Ment Health.

[90] Meza JI, Friedman LM, Dvorsky MR (2020). Outcomes of school-home intervention for attention and behavior problems: teacher adherence matters. School Ment Health.

[91] Ramer JD, Santiago-Rodríguez ME, Davis CL (2020). Exercise and academic performance among children with attention-deficit hyperactivity disorder and disruptive behavior disorders: a randomized controlled trial. Pediatr Exerc Sci.

[92] Gadow KD, Nolan EE, Paolicelli LM (1991). A procedure for assessing the effects of methlyphenidate on hyperactive children in public school settings. J Clin Child Psychol.

[93] Abikoff HB, Jensen PS, Arnold LLE (2002). Observed classroom behavior of children with ADHD: relationship to gender and comorbidity. J Abnorm Child Psychol.

[94] Miller ML, Fee VE, Jones CJ (2004). Psychometric properties of ADHD rating scales among children with mental retardation. Res Dev Disabil.

[95] Abikoff H, Hechtman L, Klein RG (2004). Symptomatic improvement in children with ADHD treated with long-term methylphenidate and multimodal psychosocial treatment. J Am Acad Child Adolesc Psychiatry.

[96] Volpe RJ, McConaughy SH, Hintze JM (2009). Generalizability of classroom behavior problem and on-task scores from the direct observation form. School Psych Rev.

[97] McConaughy SH, Harder VS, Antshel KM (2010). Incremental validity of test session and classroom observations in a multimethod assessment of attention deficit/hyperactivity disorder. *J Clin Child Adolesc Psychol*.

[98] Skansgaard EP, Burns GL (1998). Comparison of DSM-IV ADHD combined and predominantly inattention types: correspondence between teacher ratings and direct observations of inattentive, hyperactivity/impulsivity, slow cognitive tempo, oppositional defiant, and overt conduct disorder symptoms. Child & Family Behavior Therapy.

[99] DuPaul GJ, Barkley RA (1992). Situational Variability of Attention Problems: Psychometric Properties of the Revised Home and School Situations Questionnaires. J Clin Child Psychol.

[100] Dunson RM, Hughes JN, Jackson TW (1994). Effect of behavioral consultation on student and teacher behavior. J Sch Psychol.

[101] Nolan EE, Gadow KD (1994). Relation between ratings and observations of stimulant drug response in hyperactive children. J Clin Child Psychol.

[102] Thompson A V (1994). A clinical trials evaluation of a double-blinded protocol to assess the therapeutic effectiveness of stimulant medication prescribed for children ADHD.

[103] Wood JJ, Cowan PA, Baker BL (2002). Behavior problems and peer rejection in preschool boys and girls. J Genet Psychol.

[104] Austin HM (2003). Use of self-management techniques for the treatment of students diagnosed with adhd: an empirical investigation of the self-regulation of behavior.

[105] Hale JB, Fiorello CA, Brown LL (2005). Determining medication treatment effects using teacher ratings and classroom observations of children with ADHD: Does neuropsychological impairment matter?. bpsecp.

[106] Carroll A, Houghton S, Taylor M (2006). Responding to interpersonal and physically provoking situations in classrooms: emotional intensity in children with attention deficit hyperactivity disorder. Intl J Disabil Dev Educ.

[107] Luitjohan JR (2005). Validity of school-based and analog observation in assessment of attention deficit hyperactivity disorder.

[108] Simonsen BM, Bullis MD (2007). The effectiveness of using a multiple gating approach to discriminate among ADHD subtypes. J Emot Behav Disord.

[109] Riley C, DuPaul GJ, Pipan M (2008). Combined type versus ADHD predominantly hyperactive-impulsive type: is there a difference in functional impairment?. J Dev Behav Pediatr.

[110] McGoey KE, Schneider DL, Rezzetano KM (2010). Classwide intervention to manage disruptive behavior in the kindergarten classroom. J Appl Sch Psychol.

[111] Milich R, Landau S (1988). Teacher ratings of inattention/overactivity and aggression: cross-validation with classroom observations. J Clin Child Psychol.

[112] Atkins MS, Pelham WE, Licht MH (1989). The differential validity of teacher ratings of inattention/overactivity and aggression. J Abnorm Child Psychol.

[113] McNamara E, Jolly M (1990). Are disruptive behaviours reduced when levels of on-task behaviours increase? An across settings study of a class of 12- and 13-year-old Pupils—II. Behav Cogn Psychother.

[114] Bloomquist ML, August GJ, Ostrander R (1991). Effects of a school-based cognitive-behavioral intervention for ADHD children. J Abnorm Child Psychol.

[115] DuPaul GJ (1991). Parent and teacher ratings of ADHD symptoms: psychometric properties in a community-based sample. J Clin Child Psychol.

[116] Grossman J (1991). Investigation of familial and school-based risk factors for hispanic head start children.

[117] Charlebois P, LeBlanc M, Gagnon C (1993). Age trends in early behavioral predictors of serious antisocial behaviors. J Psychopathol Behav Assess.

[118] Broussard CD, Northup J (1995). An approach to functional assessment and analysis of disruptive behavior in regular education classrooms. Sch Psychol Q.

[119] Rapport MD, Denney C (1997). Titrating methylphenidate in children with attention-deficit/hyperactivity disorder: is body mass predictive of clinical response?. J Am Acad Child Adolesc Psychiatry.

[120] Murray LK (2002). Self-control training in young children.

[121] Ardoin SP, Martens BK (2004). Training children to make accurate self-evaluations: effects on behavior and the quality of self-ratings. J Behav Educ.

[122] Mclaughlin V, Gresham F, Macmillan D (2003). Comparison of teacher ratings and direct observations of children with problem behaviors.

[123] DuPaul GJ, Jitendra AK, Tresco KE (2006). Children With Attention Deficit Hyperactivity Disorder: Are There Gender Differences in School Functioning?. School Psych Rev.

[124] Hoerger ML, Mace FC (2006). A computerized test of self-control predicts classroom behavior. J Appl Behav Anal.

[125] Symons FJ, Tervo RC, Kim O (2007). The effects of methylphenidate on the classroom behavior of elementary school—age children with cerebral palsy: a preliminary observational analysis. J Child Neurol.

[126] Rapport MD, Kofler MJ, Alderson RM (2009). Variability of attention processes in ADHD: observations from the classroom. J Atten Disord.

[127] Fedewa AL, Erwin HE (2011). Stability balls and students with attention and hyperactivity concerns: implications for on-task and in-seat behavior. Am J Occup Ther.

[128] Schafer EC, Mathews L, Mehta S (2013). Personal FM systems for children with autism spectrum disorders (ASD) and/or attention-deficit hyperactivity disorder (ADHD): an initial investigation. J Commun Disord.

[129] McGrath AM, Handwerk ML, Armstrong KJ (2004). The validity of the ADHD section of the diagnostic interview schedule for children. Behav Modif.

[130] Amado-Caballero P, Casaseca-de-la-Higuera P, Alberola-Lopez S (2020). Objective ADHD Diagnosis Using Convolutional Neural Networks Over Daily-Life Activity Records. IEEE J Biomed Health Inform.

