# Supplementary material for: What types of objective measures have been used to assess core ADHD symptoms in children and young people in naturalistic settings? A scoping review
Source: BMJ Open. 2024 Sep 12;14(9):e080306. doi: 10.1136/bmjopen-2023-080306 (PMC11404249; doi:10.1136/bmjopen-2023-080306)
Supplement: online supplemental file 1 [file bmjopen-14-9-s001.pdf]

Supplementary file

Search Strategies

OVID

Electronic databases searched: MEDLINE, APAPsychINFO, Embase

((child*) OR (youth*) OR (adolescen*) OR (juvenile*) OR (teen*) OR (infant*) OR (boy*) OR (girl*) OR (student*) OR (pupil*)) AND ((ADHD) OR (“Attention deficit hyperactivity disorder”) OR ("attention deficit/ hyperactivity disorder") OR (hyperactiv*) OR (inattenti*) OR (“hyperkinetic disorder”)) AND (("Behavio* Observation*") OR (Systematic Observation*") OR ("acceleromet*") OR ("actigraph*") OR ("Direct Observation*") OR ("time sampling"))

1	Child*.mp. [mp=ti, ab, ot, nm, hw, fx, kf, ox, px, rx, ui, sy, tc, id, tm, mf, tn, dm, dv, dq]
2	limit 1 to yr="1987 -Current"
3	youth*.mp. [mp=ti, ab, ot, nm, hw, fx, kf, ox, px, rx, ui, sy, tc, id, tm, mf, tn, dm, dv, dq]
4	limit 3 to yr="1987 -Current"
5	adolescen*.mp. [mp=ti, ab, ot, nm, hw, fx, kf, ox, px, rx, ui, sy, tc, id, tm, mf, tn, dm, dv, dq]
6	limit 5 to yr="1987 -Current"
7	Juvenile*.mp. [mp=ti, ab, ot, nm, hw, fx, kf, ox, px, rx, ui, sy, tc, id, tm, mf, tn, dm, dv, dq]
8	limit 7 to yr="1987 -Current"
9	Teen*.mp. [mp=ti, ab, ot, nm, hw, fx, kf, ox, px, rx, ui, sy, tc, id, tm, mf, tn, dm, dv, dq]
10	limit 9 to yr="1987 -Current"
11	infant*.mp. [mp=ti, ab, ot, nm, hw, fx, kf, ox, px, rx, ui, sy, tc, id, tm, mf, tn, dm, dv, dq]
12	limit 11 to yr="1987 -Current"
13	boy*.mp. [mp=ti, ab, ot, nm, hw, fx, kf, ox, px, rx, ui, sy, tc, id, tm, mf, tn, dm, dv, dq]
14	limit 13 to yr="1987 -Current"
15	girl*.mp. [mp=ti, ab, ot, nm, hw, fx, kf, ox, px, rx, ui, sy, tc, id, tm, mf, tn, dm, dv, dq]
16	limit 15 to yr="1987 -Current"
17	student*.mp. [mp=ti, ab, ot, nm, hw, fx, kf, ox, px, rx, ui, sy, tc, id, tm, mf, tn, dm, dv, dq]
18	limit 17 to yr="1987 -Current"
19	pupil*.mp. [mp=ti, ab, ot, nm, hw, fx, kf, ox, px, rx, ui, sy, tc, id, tm, mf, tn, dm, dv, dq]

20	limit 19 to yr="1987 -Current"
21	2 or 4 or 6 or 8 or 10 or 12 or 14 or 16 or 18 or 20
22	"ADHD".mp. [mp=ti, ab, ot, nm, hw, fx, kf, ox, px, rx, ui, sy, tc, id, tm, mf, tn, dm, dv, dq]
23	limit 22 to yr="1987 -Current"
24	"attention deficit hyperactivity disorder".mp. [mp=ti, ab, ot, nm, hw, fx, kf, ox, px, rx, ui, sy, tc, id, tm, mf, tn, dm, dv, dq]
25	limit 24 to yr="1987 -Current"
26	"attention deficit/ hyperactivity disorder".mp. [mp=ti, ab, ot, nm, hw, fx, kf, ox, px, rx, ui, sy, tc, id, tm, mf, tn, dm, dv, dq]
27	limit 26 to yr="1987 -Current"
28	"hyperactiv*".mp. [mp=ti, ab, ot, nm, hw, fx, kf, ox, px, rx, ui, sy, tc, id, tm, mf, tn, dm, dv, dq]
29	limit 28 to yr="1987 -Current"
30	"inattentiv*".mp. [mp=ti, ab, ot, nm, hw, fx, kf, ox, px, rx, ui, sy, tc, id, tm, mf, tn, dm, dv, dq]
31	limit 30 to yr="1987 -Current"
32	"impulsiv*".mp. [mp=ti, ab, ot, nm, hw, fx, kf, ox, px, rx, ui, sy, tc, id, tm, mf, tn, dm, dv, dq]
33	limit 32 to yr="1987-Current"
34	"hyperkinetic disorder".mp. [mp=ti, ab, ot, nm, hw, fx, kf, ox, px, rx, ui, sy, tc, id, tm, mf, tn, dm, dv, dq]
35	limit 34 to yr="1987 -Current"
36	23 or 25 or 27 or 29 or 31 or 33 or 35
37	21 and 36
38	"Behavio* Observation*".mp. [mp=ti, ab, ot, nm, hw, fx, kf, ox, px, rx, ui, sy, tc, id, tm, mf, tn, dm, dv, dq]
39	limit 38 to yr="1987 -Current"
40	"Systematic Observation*".mp. [mp=ti, ab, ot, nm, hw, fx, kf, ox, px, rx, ui, sy, tc, id, tm, mf, tn, dm, dv, dq]
41	limit 40 to yr="1987 -Current"
42	"Classroom Observation*".mp. [mp=ti, ab, ot, nm, hw, fx, kf, ox, px, rx, ui, sy, tc, id, tm, mf, tn, dm, dv, dq]

43	limit 42 to yr="1987 -Current"
44	"Objective Assessment*".mp. [mp=ti, ab, ot, nm, hw, fx, kf, ox, px, rx, ui, sy, tc, id, tm, mf, tn, dm, dv, dq]
45	limit 44 to yr="1987 -Current"
46	"acceleromet*".mp. [mp=ti, ab, ot, nm, hw, fx, kf, ox, px, rx, ui, sy, tc, id, tm, mf, tn, dm, dv, dq]
47	limit 46 to yr="1987 -Current"
48	"actigraph*".mp. [mp=ti, ab, ot, nm, hw, fx, kf, ox, px, rx, ui, sy, tc, id, tm, mf, tn, dm, dv, dq]
49	limit 48 to yr="1987 -Current"
50	"Direct Observation*".mp. [mp=ti, ab, ot, nm, hw, fx, kf, ox, px, rx, ui, sy, tc, id, tm, mf, tn, dm, dv, dq]
51	limit 50 to yr="1987 -Current"
52	"time sampling".mp. [mp=ti, ab, ot, nm, hw, fx, kf, ox, px, rx, ui, an, sy, tc, id, tm, mf, tn, dm, dv, dq]
53	limit 52 to yr="1987 -Current"
54	39 or 41 or 43 or 45 or 47 or 49 or 51 or 53
55	21 and 36 and 54

EBSCOhost

Electronic databases searched: British Education Index, Education Resources Information Centre, Education Abstracts, Education Research Complete, Child Development and Adolescent Papers, Cumulative Index to Nursing and Allied Health Literature (CINAHL), Psychology and Behavioural Sciences Collection

((child*) OR (youth*) OR (adolescen*) OR (juvenile*) OR (teen*) OR (infant*) OR (boy*) OR (girl*) OR (student*) OR (pupil*)) AND ((ADHD) OR (“Attention deficit hyperactivity disorder”) OR ("attention deficit/ hyperactivity disorder") OR (hyperactiv*) OR (inattenti*) OR (“hyperkinetic disorder”)) AND (("Behavio* Observation*") OR (Systematic Observation*") OR ("acceleromet*") OR ("actigraph*") OR ("Direct Observation*") OR ("time sampling"))

S1	Child*	**Expanders** - Apply equivalent subjects<br>**Search modes** - Boolean/Phrase

S2	Child*	**Limiters** - Publication Date: 19870101-20220231<br>**Expanders** - Apply equivalent subjects<br>**Search modes** - Boolean/Phrase
S3	Youth*	**Expanders** - Apply equivalent subjects<br>**Search modes** - Boolean/Phrase
S4	Youth*	**Limiters** - Publication Date: 19870101-20220231<br>**Expanders** - Apply equivalent subjects<br>**Search modes** - Boolean/Phrase
S5	adolescen*	**Expanders** - Apply equivalent subjects<br>**Search modes** - Boolean/Phrase
S6	adolescen*	**Limiters** - Publication Date: 19870101-20220231<br>**Expanders** - Apply equivalent subjects<br>**Search modes** - Boolean/Phrase
S7	juvenile*	**Expanders** - Apply equivalent subjects<br>**Search modes** - Boolean/Phrase
S8	juvenile*	**Limiters** - Publication Date: 19870101-20220231<br>**Expanders** - Apply equivalent subjects

		**Search modes** - Boolean/Phrase
S9	teen*	**Expanders** - Apply equivalent subjects<br>**Search modes** - Boolean/Phrase
S10	teen*	**Limiters** - Publication Date: 19870101-20220231<br>**Expanders** - Apply equivalent subjects<br>**Search modes** - Boolean/Phrase
S11	infant*	**Expanders** - Apply equivalent subjects<br>**Search modes** - Boolean/Phrase
S12	infant*	**Limiters** - Publication Date: 19870101-20220231<br>**Expanders** - Apply equivalent subjects<br>**Search modes** - Boolean/Phrase
S13	boy*	**Expanders** - Apply equivalent subjects<br>**Search modes** - Boolean/Phrase
S14	boy*	**Limiters** - Publication Date: 19870101-20220231<br>**Expanders** - Apply equivalent subjects<br>**Search modes** - Boolean/Phrase
S15	girl*	**Expanders** - Apply equivalent subjects

		**Search modes** - Boolean/Phrase
S16	girl*	**Limiters** - Publication Date: 19870101-20220231<br>**Expanders** - Apply equivalent subjects<br>**Search modes** - Boolean/Phrase
S17	S2 OR S4 OR S6 OR S8 OR S10 OR S12 OR S14 OR S16	**Expanders** - Apply equivalent subjects<br>**Search modes** - Boolean/Phrase
S18	ADHD	**Expanders** - Apply equivalent subjects<br>**Search modes** - Boolean/Phrase
S19	ADHD	**Limiters** - Publication Date: 19870101-20220231<br>**Expanders** - Apply equivalent subjects<br>**Search modes** - Boolean/Phrase
S20	"Attention deficit hyperactivity disorder"	**Expanders** - Apply equivalent subjects<br>**Search modes** - Boolean/Phrase
S21	"Attention deficit hyperactivity disorder"	**Limiters** - Publication Date: 19870101-20220231<br>**Expanders** - Apply equivalent subjects<br>**Search modes** - Boolean/Phrase

S22	"attention deficit/ hyperactivity disorder"	**Expanders** - Apply equivalent subjects<br>**Search modes** - Boolean/Phrase
S23	"attention deficit/ hyperactivity disorder"	**Limiters** - Publication Date: 19870101-20220231<br>**Expanders** - Apply equivalent subjects<br>**Search modes** - Boolean/Phrase
S24	hyperactiv*	**Expanders** - Apply equivalent subjects<br>**Search modes** - Boolean/Phrase
S25	hyperactiv*	**Limiters** - Publication Date: 19870101-20220231<br>**Expanders** - Apply equivalent subjects<br>**Search modes** - Boolean/Phrase
S26	inattenti*	**Expanders** - Apply equivalent subjects<br>**Search modes** - Boolean/Phrase
S27	inattenti*	**Limiters** - Publication Date: 19870101-20220231<br>**Expanders** - Apply equivalent subjects<br>**Search modes** - Boolean/Phrase

S28	“hyperkinetic disorder”	**Expanders** - Apply equivalent subjects<br>**Search modes** - Boolean/Phrase
S29	“hyperkinetic disorder”	**Limiters** - Publication Date: 19870101-20220231<br>**Expanders** - Apply equivalent subjects<br>**Search modes** - Boolean/Phrase
S30	S19 OR S21 OR S23 OR S25 OR S27 OR S29	**Expanders** - Apply equivalent subjects<br>**Search modes** - Boolean/Phrase
S31	"Behavio* Observation*"	**Expanders** - Apply equivalent subjects<br>**Search modes** - Boolean/Phrase
S32	"Behavio* Observation*"	**Limiters** - Publication Date: 19870101-20220231<br>**Expanders** - Apply equivalent subjects<br>**Search modes** - Boolean/Phrase
S33	“Systematic Observation*"	**Expanders** - Apply equivalent subjects<br>**Search modes** - Boolean/Phrase
S34	“Systematic Observation*"	**Limiters** - Publication Date: 19870101-20220231

		**Expanders** - Apply equivalent subjects<br>**Search modes** - Boolean/Phrase
S35	"acceleromet*"	**Expanders** - Apply equivalent subjects<br>**Search modes** - Boolean/Phrase
S36	"acceleromet*"	**Limiters** - Publication Date: 19870101-20220231<br>**Expanders** - Apply equivalent subjects<br>**Search modes** - Boolean/Phrase
S37	"actigraph*"	**Expanders** - Apply equivalent subjects<br>**Search modes** - Boolean/Phrase
S38	"actigraph*"	**Limiters** - Publication Date: 19870101-20220231<br>**Expanders** - Apply equivalent subjects<br>**Search modes** - Boolean/Phrase
S39	"Direct Observation*"	**Expanders** - Apply equivalent subjects<br>**Search modes** - Boolean/Phrase
S40	"Direct Observation*"	**Limiters** - Publication Date: 19870101-20220231

		**Expanders** - Apply equivalent subjects<br>**Search modes** - Boolean/Phrase
S41	"time sampling"	**Expanders** - Apply equivalent subjects<br>**Search modes** - Boolean/Phrase
S42	"time sampling"	**Limiters** - Publication Date: 19870101-20220231<br>**Expanders** - Apply equivalent subjects<br>**Search modes** - Boolean/Phrase
S43	S32 OR S34 OR S36 OR S38 OR S40 OR S42	**Expanders** - Apply equivalent subjects<br>**Search modes** - Boolean/Phrase
S44	S17 AND S30 AND S43	**Expanders** - Apply equivalent subjects<br>**Search modes** - Boolean/Phrase

Items reported

Title of Paper, Lead Author, Year of publication, Journal Taken From, Country of Origin, Study Design, Setting(s), Do they have an ADHD diagnosis?, Type of diagnosis (If applicable) (e.g. Research or clinical), If given, sub-types of diagnosis, Eligibility criteria, Total no. of participants recruited Total no. of = participants with data ,Population Age (Mean and SD/Range), Gender [% Male (1dp)], Ethnicity (%), Study design, Aim, How was measure used?, Name of Objective Measure(s), Type of Objective Measure, If actigraph, where on body?, Brief description of Objective Measure, ADHD Core Symptom(s) Measured (I,A,H), What else it measured, Comparison measure, Duration of Measure (how long did it take? How many times per participant?", Who recorded the objective measure data?, Interobserver Agreement, Was any training given?, How was it recorded?, Validity, Reliability, Sensitivity/specifity, Type of review,

No. of databases searched, Names of databases searched, Date range of included studies, Additional searches, Dates searches conducted, Last updated search, Total no. of participants, and Eligibility criteria for review

Supplementary Table 1: Summary table of Direct Behavioural Observation Measures

	Objective Measure	Description	Lead author	Year
**1**	The Behavior Observation System for Schools (BOSS)	"The BOSS (Shapiro, 2003) provides direct observations of behavioral symptoms of ADHD in the classroom setting. Observations are segmented into 15-sec intervals and four randomly selected classroom peers provide periodic comparison data. Five BOSS behavior codes were used for the current study: Active Engaged Time (AET), Passive Engaged Time (PET), Off Task Motor (OFT-M), Off Task Verbal (OFT-V), and Off Task Passive (OFT-P)"	Hosterman	2008
**2**	Classroom Observation Code (COC)	"The Classroom Observation Code includes 10 categories with 9 of them measuring inappropriate behavior (Sattler, 1992). Behavioral categories include Interference (the child exhibits verbal or physical behaviors or noises that are disturbing to others that do not necessarily persist), Off-Task Behavior (child attends to stimuli other than the assigned work), Non-Compliance (child fails to follow teacher instruction), Minor Motor Movement (child exhibits restlessness or fidgeting), Gross Motor-Standing (child leaves his or her seat and stands on one or both legs in an erect or semi-erect position), Gross Motor-Vigorous (child leaves his or her seat in a sudden, abrupt, or impulsive manner), Out-of-Chair Behavior (the child remains out-of-chair for one full interval after the interval in which he or she first left the seat), Physical Aggression (child makes a forceful movement directed at another person, either directly or by utilizing a material object as an extension of the hand), Threat or Verbal Aggression Directed at Another Child (child uses abusive language and gestures toward another child), Threat or Verbal Aggression Directed at the Teacher (child uses abusive language and gestures toward teacher), Solicitation of Teacher (child exhibits behaviors directed at obtaining the teacher's attention), and Absence of Behavior (child exhibits no inappropriate behaviors). A total observation score was computed for each child by summing across all the behavioral categories excluding the Absence of Behavior category. In previous research,	Miller	2004

		interobserver agreement for the Classroom Observation Code averaged .80 with a range of .40 (Aggression toward a Child) to .97 (Aggression toward a Teacher). In addition, the Classroom Observation Code significantly differentiated a group of 6–12-year-old hyperactive children from a comparison group of normal children (Abikoff et al., 1980)."		
**3**	Direct Observation Form (DOF)	"The DOF is a 10-minute observation in which a trained observer examines the behavior of an identified student within a group, classroom, or recess setting and rates the student at each minute interval for on- and off-task behavior. Additionally, while observing the child, the observer writes a description of all behavior of the child. After the observation period, the observer completes a checklist of 96<br>problem items rated on a scale of 0 (behavior not observed) to 3 (definite occurrence<br>with severe intensity or occurrence lasting more than 3 minutes in duration). Achenbach<br>and Rescorla (2001) recommended that three to six 10-minute observations be averaged together in order to obtain a more representative score of the child's on-task<br>and behavioral problems. Additionally, the DOF allows for comparisons of the targeted<br>child with the behavior of control children. "	Schottelkorb	2007

**4**	The ADHD Behavior Coding System	"A behavioral catergory was coded as present following a single occurnce of the behaviour during each 30-second observation interval. A child could be scored as displaying more than one catergory of behaviour per observation interval. Percentage occurrence scores were calculated for each catergory by dividing the number of observation intervalls in which the behaviour was present by the total number of observation intervals. A total ADHD occurance score was also calculated by taking the total number of intervals coded as present across the five behavioural catergories and dividing the total number of observation intervals for all five catergories. Observations using this coding system have been founnd to be sensitive to the effects of stimulant medication and related to toehr measures of ADHD."	DuPaul	1992
**5**	The Teacher Pupil Interaction Scale (TPIS)	"This observation measure provides time-sampled observations of students' behaviors coded in one of four categories : on-task behavior, scanning behavior, social contact, and disruptive behavior . The measure provides time-sampled observations of teachers' behaviors<br>coded in one of four categories : instructing, rewarding, nonattending or neutral, and disapproving. Interrater reliability coefficients "of .80 or better" were found for the TPIS categories (Goodwin & Coates, 1977) . Because the instructing category does not distinguish between group instruction that includes the target student and one-on-one instruction, this category was subdivided into group instruction and one-on-one instruction . During each assessment period the subjects were observed by trained raters, blind to subject status, for three 15-minute periods of observation . A student and a teacher behavior code were marked every 5-second interval during these 15-minute observation periods . These observations were scheduled with the teacher and were conducted only during structured classroom instruction and independent seatwork ."	Dunson Ill	1994

**6**	The Code for Observing Social Activity (COSA)	"The direct observations in the lunchroom and on the playground are conducted using the Code for Observing Social Activity (COSA; Sprafkin, Grayson, Gadow, Nolan, & Paolicelli, 1986). The COSA was developed to evaluate aggressive and prosocial interactions between children and was found to be sensitive to stimulant drugs' effects in hyperactive children (Gadow, Nolan, Sverd, Sprafkin, & Paolicelli, 199D). With a modest amount of training, reasonably high levels of interrater reliability can be achieved (see Table 1). Each of the following behavior categories in the COSA is coded as being present or not present during 30-sec intervals: appropriate social interaction, noncompliance, nonphysical aggression, physical aggression, and play aggression."	Gadow	1991
**7**	On-/Off-Task Behavior Observation Form	"Two independent observers record data in the same sessions and then calculating the degree to which both agreed or disagreed on the intervals observed. Observations of the target and comparison children's on- and ofF-task behaviors were made two to three times a week and inter-observer agreement (or reliability) checks were conducted at least once a week. On- and off-task behaviors were recorded on the sheet listed below (see Appendix J for the actual form used) for both the target and comparison child. On-task was defined by default when no off-task behavior had been recorded during that interval Off-task was recorded when the patient engaged in either "passive" behavior (e.g., not responding to a question asked); "small motor" behavior (e.g., playing with a pencil); "large motor" behavior (e.g., getting up out of the chair); "verbal to self" (e.g., talking to oneself); "verbal to others" (e.g., talking to the person next to the subject); or a "termination" response (e.g., disrupting the classroom to such an extent that the teacher had to stop what she was doing to go to the student)."	Thompson	1994
**8**	Behavior Observation Form (BOF)	" This system used an interval, time-sampling procedure to collect data"	Kehle	1996

**9**	Behavior Assessment System for Children, Student Observation System (BASC-SOS)	Defines 65 specific target behaviours that are grouped into 13 catergories- 4 catergories of positive/adaptive behavioursand 9 of problem behaviours. 15 mins observation is divided into 30 intervals. At the end of each 30-second interval, the child's behaviour is obsrved for 3 seconds.	Brooks Mann	1997
**10**	Adapted from the Behavior Observation for Students in Schools (BOSS; Shapiro, 1996) and the Functional Assessment Observation Form (FAOF; O'Neill et al., 1997).	"Direct observation data were collected to determine the occurrence and nonoccurrence of disruptive behavior during treatment development and treatment evaluation. Data were collected using a partial interval recording system with 15-s to observe, followed by a 5-s period to record. A cassette recorder with an earpiece was used to signal each observation interval and a pencil-and-paper system was used to record observations."	Hoff	2005
**11**	"Behavioral Observation of Students in Schools (BOSS; Shapiro, 1996), adapted with School Observation Code (Gadow, Sprafkin, & Nolan, 1996) "	"Active- and passive- academic engagement was scored using a momentary time sampling procedure (15 seconds duration), and off-task behavior (verbal, motor, and passive), and noncompliance was scored using a partial interval procedure. During every 5th observation interval the same categories were coded for pre-selected classroom peers. Hence, during each measurement occasion, target student behaviors were coded for 45 intervals and the behaviors of classroom peers were coded for 12 intervals. Target student- and peer-scores for on-task and off-task categories were divided by the appropriate number of intervals to obtain target and peer scores that controlled for the number of intervals observed. Next the difference of these scores was taken to derive a measure of how much the target student differed from peers on each variable of interest. Finally, difference scores for the two on-task variables (active- and passive-academic engagement) were summed for a general score of on-task deviance, and difference scores on the three off-task variables (motor, verbal, passive) were summed for a general score of off-task deviance. It should be noted that the on-task deviance summary score is on a slightly different metric than the off-task deviance summary. This is so because although active- and passive-academic engagement are mutually exclusive, the off-task behaviors are not. Hence, a different range of possible values would be	Volpe	2003

		expected. As noted earlier, observations were performed during both mathematics and reading instruction. For each participant, the area in which the student received intervention determined the observation variables used for analysis. In some cases children received interventions in both content areas. In these cases, the content that was the target of the largest number of interventions was selected"		
**12**	Functional Observation of Classrooms and Learners (FOCAL Point)	"A computerized functional assessment program designed to facilitate direct observations of target behaviors in natural settings (Ninness, McCuller, & Ozenne, 2000). Rather than analogs, this type of functional/descriptive assessment relies exclusively on in vivo direct observations of target behaviors in natural settings. Using computer-interactive recording systems, target behaviors are recorded as they occur within particular classroom conditions. Graphs, showing the levels of target behavior that emerge under particular environmental conditions, can be developed by moving data to a spreadsheet"	Kenney	2004
**13**	A series of behaviour sampling observations (Scherer et al., 1990)	"Behaviour sampling was done using a grid on which a record was made once each minute. R's behaviour was compared to another pupil in the class, and the behaviour of both pupils was recorded using the same code. s. Using a second sweep hand, observations<br>were made in rotation at 12 second intervals, maintaining a 1 minute sampling rate for each subject"	Pester	2002

**14**	adapted from Hinshaw, Han, Erhardt, and Huber (1992) and Hinshaw (1993)	"We used a time-sampling procedure: Each observer (a) tracked children from a list of names in random order (with 5 s allotted to find each child); (b) observed each child for 5 s; and (c) recorded a behavioral code (within 3 s); then the observer moved on to the next child on the list (Hinshaw, 1993). Observers listened to a tape recording of instructions that provided the proper timing for observing and coding behavior. Of all the observations, 15% were made by two observers on the behavior of the same child at the same time. This procedure allowed us to assess interrater reliability. Observers were blind to the results of the sociometric tests and the teacher ratings, all of which were collected concurrently over the course of approximately 2 weeks"	Wood	2002
**15**	Adaptation of Barkley's (1990) "restricted academic situation coding sheet"	"Barkley's Restricted Academic Coding System has had widespread use to determine the frequency of ADHD related behaviors in the classroom (DuPaul & Stoner, 1994), and it has been used as a measure of behavioral change in the classroom to assess effects of treatment with medication (Barkley, 1998, Northrup, et al., 1997). Operational definitions for the 5 ADHD related behaviors can be seen in Table 3. One additional child behavior, "Bug-inthe-ear," was included in the coding system during the treatment phase of the study. "Bug-in-the-ear" was coded any time the student touched the bug-in-the-ear device during the coding period, and was primarily used to determine if the mechanism was a distraction for the student. Further explanation of the use of the bug-in-the-ear is included in the procedure section"	Austin	2003
**16**	an adaptation of the Restricted Academic Task (RAT; Barkley, 1990)	"The assessments took place approximately 1 to 2 hours after medication was administered. Classroom observations took place on the same day as neuropsychological testing, approximately 1 to 2 hours after the administration of the other daily dose of medication. An adaptation of the Restricted Academic Task (RAT; Barkley, 1990) was used to determine off-task, fidgeting, vocalizing, playing with objects, and out of seat behaviors. The observational procedure included a 20-second momentary time sampling technique during classroom instructional activities."	Hale	2011

**17**	Responses to Interpersonal and Physically Provoking Situations (RIPPS)	"RIPPS by way of its structured observation format was designed to provide users with a means of comparing the frequency and severity of student responses and the triggers for these. In this regard, observations that are conducted using the RIPPS employ a time-sampling procedure whereby each of two targeted students (i.e., one student with and one student without ADHD), are alternatively observed for 2 min, for a total of 20 min, within a continuous 40-min time period."	Carroll	2006
**18**	Barkley's ADHD Behavior Coding System	"A rater observed the target child engaged in independent academic work for the occurrence of five behaviors: off-task (e.g., looks away from paper), fidgets (e.g., repetitive purposeless motion), out-of-seat (e.g., buttocks leave the chair), vocalizes (e.g., inappropriate or unnecessary vocal noise), and plays with objects (e.g., touches any object in the room unrelated to the task; See Appendix A for behavioral definitions). The rater observed the child for 10 seconds and then recorded any observed behavior for 5 seconds. The length of the observation was 10 minutes for the analog situation and 30 minutes for the classroom observation (alternating observations of the target and control child in school settings; See Appendix B for Observation Recording Form)"	Luitjohan	2005
**19**	The SPA Behavior Observation Form (SPA-BOF)	"... allowed observers to record observations of students' behavior at 30-s intervals using a momentary time sampling procedure. The SPA-BOF form reflects DSM–IV criteria (e.g., fidgeting, daydreaming, other off task) and is designed to assess both a target student and a peer for the presence or absence of ADHD-related behaviors (e.g., fidgeting, daydreaming) in the classroom. Peer comparisons allow for data to be placed in context when presenting information to parents and teachers (e.g., if all students are off task, the target student's off-task behavior would appear normal). Thirty-second intervals were counterbalanced such that an observation is made of the target student or peer (nontarget student) every 15 s. The summary score reported was percentage of intervals engaged in each type of behavior."	Simonsen	2007

**20**	The Abikoff Structured School Observation Code	"Assesses off-task and disruptive behavior during structured time (e.g., circle reading activity) in the classroom. Each student was observed for a total of 15 minutes in 15-second intervals. Off-task behavior was coded using a whole interval system for sustained inattention or distractibility during the entire 15-second interval. Disruptive behavior was defined as clowning, interrupting, or talking during work. Any disruptive behavior was coded using a partial interval system (only counted once in each 15-second interval). ; Coding occurred in 15-second intervals for a total observation time of 15 minutes. Off-task was coded if the child stopped what he or she was doing or failed to engage in the task for 3 seconds or longer during a 15-second interval. Disruptive behavior included throwing objects, destroying property, yelling, etc., and was coded each time it occurred during a 15-second interval."	Ebenegger	2008
**21**	The early screening profile	"The Early Screening Profile15 was used during unstructured/ free playtime. Coding occurred in 15-second intervals for a total observation time of 15 minutes. Off-task was coded if the child stopped what he or she was doing or failed to engage in the task for 3 seconds or longer during a 15-second interval. Disruptive behavior included throwing objects, destroying property, yelling, etc., and was coded each time it occurred during a 15-second interval. Interobserver agreement was conducted on 40% of randomly selected observations and resulted in kappa coefficients of .88 for off-task and .93 for disruptive behavior."	Ebenegger	2008
**22**	The classroom observation system (COS)	"...developed by the SECC Steering Committee for the National Institute of Child Health and Development Study of Early Child Care and Youth Development (2006). The COS captured discrete child behaviours and interactions with others in the classroom over the course of two 44-minute observation cycles. Each cycles consisted of three 10-minute time sampled periods (30-second observe and 30-second record intervals)."	Kim	2010

**23**	adapted the Social Behavior Observation System of the Early Screening Procedure	"We coded the behavioral observations according to the following antisocial behavior categories: negative social engagement, off-task, disobeying established rules, and engaging in a tantrum (see Table 1 for complete definitions of targeted antisocial behaviors). The observers coded target children's behaviors using a partial interval recording system three times per week during both baseline and intervention phases. An interval time sampling procedure was used to capture an estimate of the child's behavior."	McGoey	2010
**24**	the Scope Classroom Observation Checklist (SCOC)	"The participants were observed in the classroom during a maths or literacy lesson at four, 2-min intervals, 15 min apart. The observations were made by Alison Scope. This yielded 8 min of observation data for each participant in the following categories; off-task distracted, off-task daydreaming, fidgety, out of seat, interrupting, and on-task. Three-second duration of one type of behaviour was classed as one unit of behaviour. The on-task category was then subtracted from the subtotal of the off-task categories to give a score, representing the number of attentional lapses, for each participant (mean: 136.2, SD: 78.4, range: 8–319)."	Scope	2010
**25**	Classroom Behavior Record (CBR)	"The observation consisted of a 6-sec time-sampling procedure in which appropriate and inappropriate academic and social behaviors were observed. The observers alternated between the clinicreferred boy and randomly selected male classmates during every 6-sec observation interval. At the end of each interval, the observers would enter into the Datamyte a single behavior code. If more than one behavior was manifested during an interval, the following rules of precedence were applied: In order of priority, the most (a) inappropriate, (b) recent, or (c) salient behavior was to be coded"	Milich	1988
**26**	Playroom Observations	NR	Pelham	2005

**27**	Individualized target behavior evaluation ITBE	NR	Pelham	2005
**28**	Response Class Matrix	NR	Pelham	2005
**29**	Classroom behavior code	NR	Pelham	2005
**30**	Classroom Observations of Conduct and Attention Deficit Disorders (COCADD)	"The COCADD [96] consists of 32 measures in five domains of classroom behavior (position, physical-social orientation, vocal activities, non-vocal activities, play), which are coded using a 2-s whole-interval sampling procedure. Since 1990, modified versions of the COCADD have been applied in six summer treatment program studies with ADHD children. (1) Kappa indices of IRR ranged between .42 and .78 [44] and .69 to .75 [54]. The TRR was not reported. (2) Teacher-identified students with ADHD were predicted in 83% of the cases (with 9% false positives and 24% false negatives) by using three variables of the COCADD (sitting, verbal intrusion, and talking to self) and three measures of desk checks and academic performance in the original study [96]. (3) COCADD overactive behavior correlated significantly with the IOWA Conners teacher rating of inattention-overactivity (r = .23) and COCADD verbal disruptive behavior with teacher-rated inattention-overactivity (r = .21) and aggression (r = .41) in the classroom in a sample of mixed ADHD/disruptive and unselected boys. Otherwise, no significant correlations emerged (e.g., the correlation between COCADD attending and inattention-overactivity was r = .02) [97]. (4) Sensitivity to pharmacological interventions was shown in the analogue classroom of several summer treatment program studies [53, 54, 60, 61] and a laboratory school study [62] for the modified version of the COCADD."	Minder	2018

**31**	Behavior Assessment System for Children–Student Observation System (BASC-2–SOS)	NR	Dowdy	2013
**32**	ADHD School Observation Code (ADHD-SOC)	The ADHDSOC [92] was developed on the basis of the COC [91]. It was used in one study of Table 2 [57]. The ADHD-SOC assesses interference, motor movement, noncompliance, aggression, and off-task behavior in a 15-s partial-interval time-sampling procedure. (1) IRR was acceptable (kappa = .57–.84) [57]. TRR was not specifically evaluated for the ADHD-SOC. (2) All classroom observational categories of the ADHD-SOC were shown to discriminate children with ADHD and comorbid tic disorder from controls on the group level. A combination of off-task behavior, interference, and noncompliance yielded correct identification of 91% of the subjects, but also misclassification of 20% of peers [57]. (3) There are no reports on the convergent validity of the ADHD-SOC. (4) The ADHD-SOC was sensitive to stimulant drug effects, with observed normalized classroom behavior in approximately 75% of children with ADHD and tic disorder [57].	Minder	2018
**33**	Munich Observation of Attention Inventory (MAI)	"The MAI measures off- and on-task behavior with the use of a 5-s time-sampling procedure. It was applied in one study [42] (Table 2). (1) IRR and TRR were not assessed. (2) Children with ADHD differed significantly from controls by displaying more off-task behavior, but also initiating more on-task behavior. Passive inattention explained most variance in teacher ratings. Predictive validity was not assessed [42]. (3) Observed off-task behavior was moderately related to teacher DSM-III-R ADHD ratings (r = .41–.50) and inconspicuous on-task behavior (e.g., reading, writing) reached a correlation coefficient of r = –.71 with teacher ADHD ratings [42]. (4) No treatment evaluation study has applied the MAI."	Minder	2018

**34**	Mean percentage of time spent on task	NR	Iznardo	2020
**35**	Observational coding for off-task/on-task behavior	NR	Iznardo	2020
**36**	Objective classroom observations: Average frequency count of classroom rule violations	NR	Iznardo	2020
**37**	Total duration of Attention states, Hyperactivity–impulsivity	NR	Staff	2021
**38**	ADHD total score	NR	Staff	2021
**39**	On-task percentage	NR	Staff	2021
**40**	ADHD behavior code	NR	Staff	2021

**41**	Ghent University Classroom Coding Inventory (GUCCI)	"The GUCCI is a continuous sampling coding scheme for behaviors of activity, nonsocial vocalization, and social behavior [45] or time on-task [46] (applied in two studies of Table 2). (1) IRR was high (kappa=.74–.99) [45]. TRR was not reported. (2) Significant group differences were found, but no predictive analysis of ADHD was conducted. Convergent validity (3) and sensitivity to change (4) were not evaluated"	Minder	2018
**42**	Mayes Hyperactivity Observation System (MHOS)	NR	Smith	2007
**43**	Child Behavior in Play With Parent Scale	NR	Smith	2007
**44**	No name 1	"Target children observed for 10 mins with specific codes being: (1) on-task (i.e., visual orientation to the worksheet/assigned task and/or task relevant discussion with teacher/student), (2) off-task/passive (i.e., all nontask-related activity), and (3) off-task/disruptive (i.e., audible or physically intrusive offtask behavior). "	McNamara	1990
**45**	No name 2	"Each of the targeted children was observed in an unobtrusive manner during 10-min observation periods. Three observations were performed at each of the assessment periods (baseline, post, followup). Reliability for 10-second intervals was computed for occurrence of on-task (96%), off-task/passive (91~ and off-task/disruptive (940/o) behavioral codes. The specific, nonexclusive behavior codes were (1) on-task (i.e., visual orientation to the worksheet/assigned task and/or task relevant discussion with teacher/student), (2) off-task/passive (i.e., all nontask-related activity), and (3) off-task/disruptive (i.e., audible or physically intrusive offtask behavior)."	Bloomquist	1991

**46**	No name 3	"Observations were conducted during a time when each child was completing independent seat work (e.g., math worksheet, phonics workbook). The following measures were obtained: off-task frequency, percentage of work completed (relative to classmates), percent correct of work completed, and an academic efficiency score (AES). Observations were conducted for 20-min with on-task behavior recorded for 60 consecutive intervals. Each interval was divided into 15 sec of observation followed by 5 sec for recording. A child's behavior was recorded as off-task if the child exhibited visual nonattention to written work or the teacher for more than 2 consecutive sec within each 15-sec observation interval, unless the child was engaged in another task-appropriate behavior (e.g., sharpening a pencil)"	DuPaul	1991
**47**	No name 4	"10-minute running account of each child's activities was made by an unobtrusive observer with 15-second intervals noted"	Grossman	1991
**48**	No name 5	"Observations were conducted when each child was completing independent desk work (e.g., math worksheet anld phonics workbook)"	DuPaul	1992
**49**	No name 6	"Two categories (task-appropriate, task-inappropriate) were added to the coding system in order to describe the child's interest in the task"	Charlebois	1993
**50**	No name 7	"A 20-s partial interval observation procedure was used to record the occurrence or nonoccurrence of all student target behaviors and independent variables during all observations. All classrooms observations and functional analysis conditions lasted 10 m"	Broussard	1995
**51**	No name 8	"Each interval was divided into 15 second s of observation followed by 5 seconds for recording. A child' s behavio r was categorized as either on- or offtask in a manner identi cal with that used by Rapport et al. (1987). Off-task behavior was defined as visual inattention to one's mater ials for more rhan 2 co nsecutive seco nds wirhi n each 1S-seco nd observa - rion int erval. unl ess the child was engage d in an alternative taskappropriare beha vior (e.g.. sharpening a pencil). "	Rapport	1996

**52**	No name 9	"1). It has both School and Home versions, thereby allowing for valid cross-contextual<br>comparisons of level of "impairment". This aspect has clear implications with regard to<br>the DSM-IV criterion that there must be evidence of functional impairment in two<br>settings (i.e., home and school).<br>2). It was constructed with the DSM-IV criteria explicitly in mind and the device also has<br>a "DSM-IV" form that allows each of the rated items to be linked to each diagnostic<br>criterion.3). The scales allow for objective "frequency" ratings of each behavior (e.g., "One to<br>several times per hour") and therefore do not require the respondent to engage in<br>speculation or interpretation regarding the "metric" of the question"	Yelich	2001
**53**	No name 10	"All observations were made between 9 A.M. and noon, and only made during formal lessons when the teacher was in the room."	Brewis	2002
**54**	No name 11	"Trained graduate and undergraduate researchers performed behavioral observations for each individual child for one 6-minute time period per week dining “carpet time” when the children were sitting and listening to a story. Observations were performed using 30-second partial interval recording based on three different behaviors commonly described as impulsive. A second observer was present for at least 20% of the sessions to assess interobserver reliability."	Murray	2002
**55**	No name 12	"Observations were conducted during situations in which the students were required to sustain attention to a task without adult assistance, which has been noted in the literature as times when children with ADHD have difficulty maintaining appropriate behavior (Barkley, Fischer, Newby, & Breen, 1988). Alterations were made to the behavioral definitions developed by Barkley et al., in order to increase the likelihood that students could learn to accurately evaluate their behavior in comparison to direct observations"	Ardoin	2004

**56**	No name 13	"They started the stopwatch every time the student engaged in the target behavior and stopped the watch when the student was no longer engaging in the target behavior. The total time was taken from the stopwatch after the 10 min. Each target behavior was observed independently from each other. Observations were collected in May and June 2002, during Year 2 of the original project."	McLaughlin	2003
**57**	No name 14	"Direct observation data were collected to determine the occurrence and nonoccurrence of disruptive behavior during treatment development and treatment evaluation. Data were collected using a partial interval recording system with 15-s to observe, followed by a 5-s period to record. A cassette recorder with an earpiece was used to signal each observation interval and a pencil-and-paper system was used to record observations"	DuPaul	2006
**58**	No name 15	"Data on classroom behavior were collected for 3 consecutive mornings for each pair of children using an audiotape player with headphones that indicated 10-s intervals. The observers sat in a corner of the room and collected data between 9:00 a.m. and 12:00 p.m. Data were collected only during lesson time (when the children were assigned seatwork or when the teacher was giving verbal instructions). Data were not collected when either child was receiving one-to-one attention from an adult, during the children's play time, during classroom transitions, or when the observers could not see both of the children."	Hoerger	2006
**59**	No name 16	"A direct observation protocol based on a 10-second partial interval (1/0) for stereotyped and disruptive behavior and time sampling for task-related behavior was used to code data collected directly from each child's classroom for two 30-minute sessions on 2 separate days for each week of the trial"	Symons	2007

**60**	No name 17	"A trained undergraduate- or graduate-level research assistant observed each child for 60 consecutive intervals during each observation day throughout the study. Each interval was divided into 15 s of observation followed by 5 s for recording. A child's behavior was categorized as either on or off task using a partial interval schedule. The recent meta-analytic review by Kofler et al. (2008) found no difference between studies defining off task based on partial or whole intervals. Off-task behavior was defined as visual inattention to one's materials for more than 2 consecutive seconds within each 15-second observation interval, unless the child was engaged in an alternative task-appropriate behavior (e.g., sharpening a pencil). An observer was situated in each classroom such that they (a) avoided direct eye-to-eye contact with observed children, (b) were distanced from them by approximately half the classroom size, and (c) maintained a clear view of the child to allow determination of task-related attention. Observers were blind to children's diagnostic standing"	Rapport	2009
**61**	No name 18	"The Fixed Interval Sampling (FIS) technique involved close observation of Carl, usually over the course of a lesson, and the recording of his predominant behaviour over 15-second periods. It was possible to use FIS in almost any setting over periods of varying duration. When using the Instantaneous Time Sampling (ITS) technique, a 'snapshot' of the behaviour displayed by both Carl and the comparison pupil was recorded at 30-second intervals. At the beginning of the case study, the class teacher was asked to identify a same-sex classmate as 'typical' or 'average' as a comparison (DuPaul and Stoner 2003). Observations were carried out over 10-minute periods (providing a 'score' out of 20), but often three 10-minute periods were recorded in one lesson, in order to identify any patterns of behaviour."	Wheeler	2009
**62**	No name 19	"The children whose ADHDT score was at or above the 92nd percentile were observed using MTS (Rapp et al., 2008), wherein every 30 s, the observer would code the student's behavior on the basis of several behavioral classifications. Observers carried a stopwatch to record the 30-s time interval and marked their observations on each child's designated worksheet. This design was chosen because MTS at 30 s has beenshown to reduce the number of false positives for duration events (Rapp et al., 2008). In effect, this MTS interval	Fedewa	2011

		would serve to make the observations more valid and representative of the child's behavior throughout the baseline and observation periods"		
**63**	No name 20	"...the primary investigators observed participants in the private school classroom to determine typical behaviors. Following this informal observation, the most frequent observed behaviors were defined and assigned a behavior code (Table 2). Direct observation data sheets were then created by the investigators to record on-task versus off-task behaviors of the 11 participants during each 45-min observation period per session. The observation session was divided into 77, 30-s intervals, and participants with ASD and ADHD were randomly assigned to an observation sequence to control for order effects. This system allowed for seven recorded behaviors for each participant per day"	Schafer	2013
**64**	No name 21	"...four 20-min observations were conducted with peer comparisons in his core academic classes utilizing partial interval time sampling. The observer did not interact with Dean until after the observations to minimize the impact on his classroom behaviors. This allowed the evaluator to observe Dean's behaviors in his natural environment and consider any environmental or peer contingency variables. In each classroom setting, instruction was delivered in a structured and well-organized manner with appropriate visual aids. Students were provided concise directions on assignment expectations, the teachers were engaging, and opportunity was afforded for student questions and participation. No notable peer contingency disruptions were indicated."	Parker	2016
**65**	No name 22	"Data collection sessions lasted 20 min and were separated into 20-s intervals. The researchers looked up to observe student behavior when the iPad signaled the end of each interval, and recorded whether the student was on task based on the definition used in the study. Students were always observed in the same order (Allison, then Carl, and then Ben) for each interval, and all three students were observed in every interval"	Aspiranti	2021

Supplementary Table 2: Summary table of Acceleration-sensitive Devices

**1**	Motionlogger actigraph BASIC model, Ambulatory Monitoring Inc., PO Box 609, Ardsley, NY 1050	"ZCM counts the number of excursions the device's motion sensor signal, bandpass-filtered between 2 and 3 Hz and sampled at 10 Hz, makes over the factory-calibrated threshold, which is equivalent to 0.02 g at midband. This mode of operation provides a frequency-of-movement measure. A 1-minute epoch or storage rate was used, and stored epoch values were averaged over the carefully controlled and predefined 20-minute periods when the children were in the three situations (i.e., the classroom Seatwork, classroom Groupwork, and playground Recess settings)."	Swanson	2002
**2**	ActiTrac activity monitor	"The device is an ambulatory motion recorder that looks like a digital wristwatch and contains a biaxial accelerometer sensor that is able to record motion in two planes. Data are recorded each minute in calibrated "milli-g" units of acceleration. ActiTracs were worn on the dominant wrist during regular classroom situations throughout the school day for 3 consecutive school days. Data from active periods such as gym, recess, or lunch were removed from the analyses"	McGrath	2004
**3**	BuzzBee®	"A prototype of the BuzzBee® (Ambulatory Monitoring Inc.,Ardsley, NY) feedback actigraph that measured 2.75 1.69 0.625 inches and weighed 2.5 ounces with its 1/2 AA lithium battery that powers the data collection and feedback was used. The actigraph technology used in the BuzzBee® was derived from AMI's Mini Motionlogger ® BASIC model. A piezoelectric sensor generates a voltage that is directly proportional to the rate of change of movement; positive voltages are associated with acceleration, negative voltages with deceleration. This signal is pass-band filtered in the 2–3 Hz range with a sensitivity of .02 g at mid-band to remove artifact. Movement frequencies higher than 3 Hz and lower than 2 Hz probably derive from sources other than the participant's behavior such as holding on to a vibrating object. The resulting voltages are rectified (the absolute value taken) and integrated (summed) over 0.1-second intervals resulting in measurement units of volt-seconds.1 It is important to note that resulting Activity Units are directly proportional to movement intensity and form a ratio scale with absolute zero and equal unit of		

		measurement up to the saturation point of the device. Vibratory feedback regarding current behavior was provided by the same type of"		
**4**	Mini Motionloggers from Ambulatory Monitoring Inc	The actigraphs used in the study were MiniMotionloggers from Ambulatory Monitoring Inc. A Mini-Motionlogger is a small wristwatch-like device to be worn continuously on the non-dominant wrist. This device is capable of detecting accelerations (greater than 0.01 g) caused by subtle movement and of transducing the accelerations into the voltage.The data were collected in the mode, which measures movement frequency by recording a count each time the filtered voltage crosses a defined threshold voltage regardless of whether the voltage is increasing or decreasing (zero crossing mode). It also allows daytime activity counts to be recorded for each 1-minute epoch. The threshold voltage was set at the default setting (mode 18).	Tsujii	2007
**5**	Micro miniMotionloggers from Ambulatory Monitoring Inc	It was expected that children would wear this device continuously throughout the study period. However, they were instructed to remove it when either bathing, or during rigorous exercise, or when performing any activity that might result in the device being damaged. They were also informed that they should just lead their lives normally while wearing the actigraph device	Ogino	2018
**6**	Actitrac activity monitor	"The device is an ambulatory motion recorder that looks like a digital wristwatch and contains a biaxial accelerometer sensor that is able to record motion in two planes. Data are recorded each minute in calibrated “milli-g” units of acceleration. ActiTracs were worn on the dominant wrist during regular classroom situations throughout the school day for 3 consecutive school days.	McGrath	2004

		Data from active periods such as gym, recess, or lunch were removed from the analyses"		
**7**	The Computer Science and Applications, Inc. (CSA)/Manufacturing Technology, Inc. (MTI) actigraph	"The actigraphs measured activity level 10 times per second over 1-minute epochs resulting in 600 measurements per minute. The average activity level for each minute was stored for each of the 1,440 minutes per 24 hr."	Licht	2009
**8**	actigraph (LIG Nex1 Co., Ltd., South Korea, 2007)	"Activity data was collected for 1-3 days, for 3 hours per day, during school hours. An actigraph (LIG Nex1 Co., Ltd., South Korea, 2007) was placed on the non-dominant wrist of each child from the beginning of the first lesson to the end of the final lesson. 3-axial acceleration data were recorded in a built-in memory chip at a frequency of 32 Hz."	Kam	2010
**9**	Actical activity accelerometer (Actical, Version 2.0, Mini Mitter, Respironics)	"Activity was recorded in 30 s epochs for total daily step counts and energy expenditure calculated for each day. Data collection took place between February and mid June 2009. Children with DCD and healthy controls were tested within 2 weeks in order to limit variation in movement that could be influenced by seasonal differences in physical activity and accessibility to physical activity."	Baerg	2011
**10**	Actiheart	"Each time an actigraph is moved compared to a reference signal a voltage is generated. The number of movements during a 1 min interval is accumulated and saved before resetting the counter to zero, providing a measure of activity frequency (counts/min). Through an interface, the actigraph data were loaded into a software program (Sleep Analysis, version 7, Cambridge Neurotechnology Ltd, Cambridge, UK) for further analysis."	Imeraj	2011

**11**	GT1 M, Actigraph, Florida)	"Participants were instructed to wear the accelerometers around the hip during 5 consecutive days. Validity was defined as at least 3 days of recording (2 weekdays and 1 day on the weekend),30 with a minimum of 6 hours per day. The validity of wearing the accelerometer for 6 hours per day was highly correlated with 10-hour validity in this population (n ¼ 502, r ¼ .92, P < .001, unpublished observations). Data from monitored days were extrapolated to get a daily average by weighing weekdays and weekends [(5 þ 2)/7]. Sequences of at least 10 minutes of consecutive zero values were removed and interpreted as accelerometer not worn.31 Total physical activity was expressed as counts per minute, divided by mean daily wearing time of 10.8 + 1.2 hours. Time spent in moderate-vigorous, vigorous, and sedentary activity was defined as the number of epochs (15-second intervals) per hour spent in activities of 420 counts, 842 counts, and 25 counts, respectively.29,32"	Ebenegger	2011
**12**	Actiwatch 4	"...(37 mm × 29 mm × 10 mm), light-weight (16 g) actigraphs (Actiwatch 4, Philips Respironics, Murrysville, PA, United States), detecting the highest movement-induced accelerations (0.5–7.0 Hz) during 15-s intervals, generating a transient voltage signal proportionate to the acceleration rate (Chen and Bassett, 2005; Cambridge Neurotechnology, 2008)"	Veenman	2017
**13**	Runscribe™ inertial sensors	"The sensors used were two Runscribe™ inertial sensors (Scribe Labs, San Francisco, CA, USA)<br>containing a tri-axial accelerometer which were applied to the wrist and ankle of the dominant arm<br>and leg by aligning their long axis with limb and placing them within an elasticated 'sweat' band,<br>see Figure 1. The sampling frequency for each sensor was set at 10 Hz with the addition of a low pass<br>anti-aliasing filter at 5 Hz. The relatively low sampling frequency was chosen to allow 24 h collection<br>of raw accelerometer data: this is expected to adequately characterize the movement patterns as<br>the large majority of the power in voluntary human arm movement is below 2 Hz [25], although<br>there may be low amplitude movements up to 8.4 Hz for occasional maximal	Muñoz-Organero	2019

		speed movements [26].<br>Synchronization of sensors was through simultaneous initialization. The sensors' measurement range<br>was 16 g, which was sufficient to avoid saturation (confirmed by inspection of the collected data)"		
**14**	The Actiwatch AW64	"The Actiwatch AW64 (Cambridge Neurotechnology Ltd, UK) was used to objectively assess sleep and CAR. Using the software Actiwatch Activity and Sleep Analysis 5 (version 5.32), each epoch was scored as sleep or wake according to the algorithm proposed by Oakley [20], setting the wake sensitivity to low [21,22]. The same software was used to extract minute-by-minute raw motor activity counts across the 24 h, in order to draw the raw CAR"	Tonetti	2019
**15**	ActiGraph GT3x device	"Actigraphic signals were acquired with the ActiGraph GT3x device [36], placed on the dominant wrist of each patient. This device measures the acceleration on each of the three Cartesian axes, registering a sample every second (operating frequency is fs = 1Hz). The aggregate signal r = ||(x, y, z)|| = (x2 + y2 + z2) will be the measurement used as the input signal, i.e., a 1D signal is used per patient. The actual measurement is a count [37], as provided by the commercial device."	Amado-Caballero	2020
**16**	Tiny actigraph, Cambridge Neurotechnology Version 2.56	"Konrad et al. 2005...Tiny actigraph, Cambridge Neurotechnology Version 2.56...Preferred wrist First 90 min of each school day and 80 min of neuropsychological assessment...0, 25 min epoch"	De Crescenzo	2014

**17**	Pensacola FL	"...participants were sent home with an accelerometer (Actigraph GT3X, Pensacola FL) and asked to wear it over their right hip for seven consecutive days, only removing it for sleep and/or prolonged water activities (e.g., bath or pool). A logbook was sent home with the primary caregiver to record the times when the accelerometer was taken of and placed back on the child. Non-wear periods were determined and subsequently removed from analysis using the information from the logbook as well as any periods of≥60 min of consecutive zero counts. A day of wear was considered to be valid if the child had≥10 h of wear time and only children with at least three valid days were included in the analyses. Three second epochs were used to analyze the data and Evenson cut points were applied to determine the average daily minutes of MVPA [50]. As the Evenson cut points were created using 15 s epochs, it was divided by fve and applied to each three second epoch. The total time spent in MVPA was calculated for each day and averaged across all valid days of wear. All accelerometer data were cleaned and processed using Actilife Software (Actigraph, Pensacola Fl.)"	James	2021
**18**	Basic mini-motionlogger actigraph, AMI Inc	"These actigraphs employ a piezoelectric beam sensor, with a fixed sensitivity of 2–3 Hz to detect accelerations greater than 0.1 G. With a 32 K memory and a sampling rate of 10 Hz, data can be collected for up to 22 days after initialization and downloaded in zerocrossing (ZC) mode through an auto-interface into ACT operational software."	Corkum	2007

**19**	No name actigraphy 1	"The rest-activity rhythm was assessed using actigraphy, 33 for 3 periods: 5 days before treatment (pre) during which the children were medication free, 5 days immediately following a 6-week treatment period (post), and again 5 days after a treatment-free period of 6 weeks (delayed). On each occasion, the child wore an actigraph around the right wrist. The actigraph registers arm movements. From the resulting rest-activity rhythms, 5 variables were calculated: 1) The interdaily stability (IS) quantifies the strength of coupling between the rest-activity rhythm and supposedly stable zeitgebers. In normal cases, the activity patterns of individual days resemble each other very much, whereas days may differ considerably with rhythm disturbances. 2) The intradaily variability (IV) quantifies the fragmentation of the rhythm, that is, the frequency and extent of transitions between rest and activity. In normal cases, one has a major activity period during the day and a major inactivity period during the night, whereas brief alternating bouts of rest and activity are characteristic of rhythm disturbances. 3) The relative amplitude (RA) quantifies the difference between the main activity (day) and rest (night) periods. 4) M10 reflects 10 h of the child's maximum activity within 24 h. 5) L5 represents the 5 least active hours within 24 h. In normal cases, the daytime activity is high and the nighttime activity is low, resulting in high amplitude. With circadian rhythm disturbances, nighttime activity may increase, whereas daytime activity may decrease resulting in a low amplitude."	Jonsdottir	2004

Supplementary Table 3: Details of included Direct Behavioural Observations studies

Objective Measure	First Author	Year	Duration of measure	Intervals	Training	Details on training	Who coded	Format of recording
The Behavior Observation System for Schools (BOSS)	Stevens	1998	10 mins	-	Yes	-	S	-
	Volpe	2003	-	-	-	-	-	-
	DuPaul	2004	-	15-secs	-	-	-	-
	Hoff	2005	45 mins	15-secs	Yes	-	S	P
	Vile Junod	2006	15 mins	15-secs x 60	Yes	-	S	-
	Hosterman	2008	-	15-secs	-	-	-	-
	Vile Junod	2006	15 mins	15-secs x 60	-	-	-	C
	Steiner	2011	-	-	-	-	-	-
	Pfiffner	2013	15 mins	-	-	-	R	-
	Steiner	2014	15 mins	-	-	-	R	-
	Steiner	2014	15 mins	15-secs x60	-	-	R	-
	Steiner	2014	15 mins x 3	-	Yes	-	R	-
	Slattery	2016	-	-	Yes	Yes	R	-
	Simpson	2016	-	-	Yes	-	R	I
	Kennerley	2018	30 mins	x120	Yes	-	R	-
	Minder	2017	-	-	Yes	Yes	-	-
	Jiang	2019	-	15-secs	Yes	-	-	-
	Meza	2020	-	15-secs	Yes	-	C	-
	Ramer	2020	-	15-secs	-	-	S	-
	Dowdy	2013						

	Kofler	2008						
	Minder	2018						
	Staff	2021						
Classroom Observation Code (COC)	Gadow	1991	90 mins x 2	15- secs	Yes	-	R	-
	Abikoff	2002	16 mins	15-secs x 16 x 4	Yes	-	-	-
	Miller	2004	12 mins	15-secs x 12 x 4	Yes	-	R	-
	Veenman	2017	8 mins x 2	15-secs	Yes	-	-	-
	Minder	2018						
	Staff	2021						
	Kofler	2008						
	Abikoff	2004	-	-	Yes	-	-	-
	Stevenson	2010	8 mins x 3	-	-	-	-	-
	Tiah	2013	30 mins	15-secs	-	-	-	-
Direct Observation Form (DOF)	Schottelkorb	2007	10 mins x 3	1 min	Yes	-	R	-
	Schottelkorb	2009	10 mins	-	-	-	R, S	-
	Volpe	2009	10 mins	-	-	-	-	-
	McConaughy	2010	10 mins x 3/4	-	Yes	-	-	-
	Johnson	2020	x4	-	Yes	-	-	C
	Kofler	2008						
	Dowdy	2013						
	Minder	2018						
	Staff	2021						
	Montague	1994						
The ADHD Behavior Coding System	DuPaul	1992	15-20 mins	30-secs	-	-	R	-

The Teacher Pupil Interaction Scale (TPIS)	Dunson Ill	1994	15 mins	5-secs	Yes	-	-	-
the Code for Observing Social Activity (COSA)	Nolan	1994	-	15 or 30-secs	Yes	-	-	-
	Gadow	1991	90 mins x 2	30-secs	Yes	-	R	-
On-/Off-Task Behavior Observation Form	Thompson	1994	-	-	-	-	-	-
Behavior Observation Form (BOF)	Kehle	1996	-	-	-	-	-	-
Behavior Assessment System for Children, Student Observation System (BASC-SOS)	Brooks man	1997	15 mins	30-secs	-	-	-	-
	Bianco	1997	15 mins	3-secs	-	-	School psychologist	-
Adapted from the Behavior Observation for Students in Schools (BOSS; Shapiro, 1996) and the Functional Assessment Observation Form (FAOF; O'Neill et al., 1997).	Hoff	2005	45 mins	15-secs	Yes	-	S	P
"Behavioral Observation of Students in Schools (BOSS; Shapiro, 1996), adapted with School Observation Code (Gadow, Sprafkin, & Nolan, 1996) "	Volpe	2003	-	-	-	-	-	-
Functional Observation of Classrooms and Learners (FOCAL Point)	Kenney	2004	15 mins x 3	30-secs	Yes	-	First author and two observer	C
A series of behaviour sampling observations (Scherer et al., 1990)	Pester	2002	30 mins x 2	-	-	-	-	-

adapted from Hinshaw, Han, Erhardt, and Huber (1992) and Hinshaw (1993)	Wood	2002	-	-	-	-	-	-
Adaptation of Barkley's (1990) "restricted academic situation coding sheet"	Austin	2003	-	-	-	-	-	-
an adaptation of the Restricted Academic Task (RAT; Barkley, 1990)	Hale	2005	-	20 secs	Yes	Yes	R	-
Responses to Interpersonal and Physically Provoking Situations (RIPPS)	Carroll	2006	-	-	-	-	-	-
	Minder	2018						
Barkley's ADHD Behavior Coding System	Luitjohan	2005	30 mins	15-secs	-	-	-	-
The SPA Behavior Observation Form (SPA-BOF)	Simonsen	2007	-	30-secs	-	-	The first author and trained behavioral observer	-
The Abikoff Structured School Observation Code	Riley	2008	15 mins	15-secs	-	-	S	-
The early screening profile	Riley	2008	15 mins	15-secs	-	-	S	-
The classroom observation system (COS)	Kim	2010	10 mins x 3	30-secs	-	-	-	-
adapted the Social Behavior Observation System of the Early Screening Procedure	McGoey	2010	20- 30 mins	15-secs	Yes	-	S	-
the Scope Classroom Observation Checklist (SCOC)	Scope	2008	8 mins	2 mins	Yes	-	R	-
	Scope	2010	8 mins	2 mins	-	-	-	-
Classroom Behavior Record (CBR)			16-24 mins	6-secs	Yes	40 hours	Teachers	Datamyte calculator

Playroom Observations (Milich et al., 1982, 1986)	Pelham, JR	2005	-	-	-	-	-	-
Individualized target behavior evaluation ITBE	Pelham, JR	2005	-	-	-	-	-	-
Response Class Matrix	Pelham, JR	2005	-	-	-	-	-	-
Classroom behavior code	Pelham, JR	2005	-	-	-	-	-	-
Classroom Observations of Conduct and Attention Deficit Disorders (COCADD)	Pelham, JR	2005	-	-	-	-	-	-
Behavior Assessment System for Children–Student Observation System (BASC-2–SOS)	Dowdy	2013	-	-	-	-	-	-
ADHD School Observation Code (ADHD-SOC)	Minder	2018	-	15-secs	-	-	-	-
Munich Observation of Attention Inventory (MAI)	Minder	2018	-	5-secs	-	-	-	-
Mean percentage of time spent on task	Iznardo	2020	-	-	-	-	-	-
Observational coding for off-task/on-task behavior	Iznardo	2020	-	-	-	-	-	-
Objective classroom observations: Average frequency count of classroom rule violations	Iznardo	2020	-	-	-	-	-	-
Total duration of Attention states, Hyperactivity–impulsivity	Staff	2021	20 mins	2 mins	-	-	-	-
On-task percentage	Staff	2021	-	-	-	-	-	-
ADHD behavior code	Staff	2021	15 mins x 1	-	-	-	-	-
Ghent University Classroom Coding Inventory (GUCCI)	Staff	2021	60 minutes x	-	-	-	-	-

			4 over 2 days						
Mayes Hyperactivity Observation System (MHOS)	Smith	2007	-	-	-	-	-	-	
Child Behavior in Play With Parent Scale	Smith	2007	-	-	-	-	-	-	
No name 1	McNamara	1990	5 min	10 secs	Yes	-	S	-	
No name 2	Bloomquist	1991	10 min	10 secs	Yes	Yes	S	-	
No name 3	DuPaul	1991	20 min	15 secs	Yes	-	R	-	
No name 4	Grossman	1991	10 min	15 secs	-	-	R	-	
No name 5	DuPaul	1992	20 min	15 secs	-	-	R	-	
No name 6	Charlebois	1993	4 hours	15 secs	Yes	Yes	-	Computerized Event Recorder	
No name 7	Broussard	1995	10 min	20 secs	Yes	-	S	-	
No name 8	Rapport	1996	20 min	15 secs	-	-	S	-	
No name 9	Yelicj	2001	30 min	30 secs	-	-	Blinded observer	-	
No name 10	Brewis	2002	2 min	-	-	-	-	C	
No name 11	Murray	2002	6 min	30 secs	Yes	-	R	-	
No name 12	Ardoin	2004	10-15 min	10 secs	Yes	-	-	-	
No name 13	McLaughlin	2003	10 min	-	Yes	-	R	-	
No name 14	DuPaul	2006	45 min	15 secs	-	-	R	P	
No name 15	Hoerger	2006	-	10 secs	Yes	-	S	Audiotape player with headphones	
No name 16	Symons	2007	30 mins	10 secs	Yes	-	-	Handheld digital camera	
No name 17	Rapport	2009	20 mins	15 secs	Yes	-	R	-	
No name 18	Wheeler	2009	10 mins	15 secs	-	-	-	-	
No name 19	Fedewa	2011	30 mins	30 secs	Yes	Yes	S	P	

No name 20	Schafer	2013	45 mins	30 secs	Yes	-	R	-
No name 21	Parker	2016	20 mins	-	-	-	-	-
No name 22	Aspiranti	2021	20 mins	20 secs	Yes	-	S	C

S= student; R=researcher; P=pencil and paper; C=computer

Supplementary Table 4: Details of included Acceleration-sensitive measures studies

Objective Measure	First Author	Year	Duration of measure	Intervals	Training	Details on training	Who coded	Format of recording
Motionlogger actigraph BASIC model, Ambulatory Monitoring Inc., PO Box 609, Ardsley, NY 1050	Swanson	2002	-	-	-	-	-	-
Motion Logger Actigraph	Jensen	2004	-	-	-	-	-	-
MicroMini-Motionlogger actigraphs (Ambulatory Monitoring Inc., Ardsley, N.Y.)	Kopstein	2004	-	-	-	-	-	-
ActiTrac activity monitor	McGrath	2004	3 days	-	-	-	-	-
the Computer Science and Applications, Inc. (CSA) actigraph	Licht	2005	2 weeks	-	-	-	-	-
BuzzBee®	Tryon	2006	30 min	-	Yes	-	T	-
AMI Mini	Tryon	2006	30 min	-	Yes	-	T	-
Mini Motionloggers from Ambulatory Monitoring Inc	Tsujii	2007	-	-	-	-	-	-
Motionlogger® BASIC	Corkum	2007	1 week	-	-	-	-	C
Micro miniMotionloggers from Ambulatory Monitoring Inc	Ogino	2018	> 7 days	-	-	-	-	-
Actitrac device individual monitoring system	De Crescenzo	2014	-	-	-	-	-	-
The Computer Science and Applications, Inc. (CSA)/Manufacturing	Licht	2009	2 weeks	-	-	-	-	-

Technology, Inc. (MTI) actigraph								
Mini motionloggers	Tsujii	2009	1 week	-	-	-	-	-
actigraph (LIG Nex1 Co., Ltd., South Korea, 2007)	Kam	2010	1-3 days, for 3 hours per day, during school hours	-	-	-	-	C
Actical activity accelerometer (Actical, Version 2.0, Mini Mitter, Respironics)	Baerg	2011	7 days	-	-	-	-	-
Actiheart	Imeraj	2011	(3 school days and 2 weekend days)	-	-	-	-	-
	Casaseca-de-la-Higuera	2012	24 hours	-	-	-	-	-
LIG Nex1 Co., Ltd., Yongin, Korea	Kam	2011	3 hours	-	-	-	R	-
GT1 M, Actigraph, Florida)	Ebenegger	2011	5 days	-	-	-	-	-
	Lin	2013	7 days	-	-	-	-	-
minimotionlogger or motionlogger watch	Faedda	2016	3–5 days during the school week	-	-	-	Skilled technician	-
Actiwatch 4	Veenman	2017	7 days	-	-	-	-	-
Runscribe™ inertial sensors	Muñoz-Organero	2019	24 hours	-	-	-	-	-

The Actiwatch AW64	Tonetti	2019	5 days	-	-	-	-	-	-
ActiGraph GT3x device	Amado-Caballero	2020	-	-	-	-	-	-	-
	James	2021	7 days	-	-	-	-	-	-
Tiny actigraph, Cambridge Neurotechnology Version 2.56	De Crescenzo	2014	-	-	-	-	-	-	-
Actigraph, Ambulatory monitoring inc	De Crescenzo	2014	-	-	-	-	-	-	-
Pensacola FL	James	2021	7 days	-	-	-	-	-	-
Actitrac device individual monitoring system	De Crescenzo	2014	-	-	-	-	-	-	-
NR	De Crescenzo	2014	-	-	-	-	-	-	-
Basic mini-motionlogger actigraph, AMI Inc	De Crescenzo	2014	-	-	-	-	-	-	-
No name 1	Jonsdottir	2004	5 days	-	-	-	-	-	-

S= student; R=researcher; T=teacher, P=pencil and paper; C=computer,
