# Supplementary material for: What types of objective measures have been used to assess core ADHD symptoms in children and young people in naturalistic settings? A scoping review
Source: BMJ Open. 2024 Sep 12;14(9):e080306. doi: 10.1136/bmjopen-2023-080306 (PMC11404249; doi:10.1136/bmjopen-2023-080306)
Supplement: Supplementary file 2 [file bmjopen-2023-080306.reviewer_comments.pdf]

PEER REVIEW HISTORY

BMJ Open publishes all reviews undertaken for accepted manuscripts. Reviewers are asked to complete a checklist review form and are provided with free text boxes to elaborate on their assessment. These free text comments are reproduced below.

ARTICLE DETAILS

**TITLE (PROVISIONAL)**	What types of objective measures have been used to assess core ADHD symptoms in children and young people in naturalistic settings? A scoping review
**AUTHORS**	Kelman, Charlotte; Thompson Coon, Jo; Ukoumunne, Obioha; Moore, Darren; Gudka, Rebecca; Bryant, Eleanor; Russell, Abigail

VERSION 1 - REVIEW

**REVIEWER NAME**	***Ortiz, Mercedes***
**REVIEWER AFFILIATION**	University of Washington, Psychiatry & Behavioral Sciences; Seattle Children's Hospital
**REVIEWER CONFLICT OF INTEREST**	Na
**DATE REVIEW RETURNED**	06-Dec-2023

**GENERAL COMMENTS**	Interesting scoping review. Review and ensure unit reporting consistencies. For example, in some sections (Results and Characteristics of sources, some data is reported with n units and some with or without percentages).<br>Methods - Descriptive statistics were used to analyses included papers (were descriptive statistics used on the 2801 papers identified or the 128 included studies). This makes it sound like methods were used on the 2801 larger n than 128.

**REVIEWER NAME**	***Salmi , Juha***
**REVIEWER AFFILIATION**	University of Turku
**REVIEWER CONFLICT OF INTEREST**	Na
**DATE REVIEW RETURNED**	12-Mar-2024

GENERAL COMMENTS

General comment

Manuscript by Kelman and colleagues reports results from a scoping review examining the opportunities of objective measures in diagnostic assessment of ADHD. The topic of the study is timely and important and the search and study selection process appears to follow the required quality standards. However, the framing of the study and description of the background, limited depth of the analysis, as well as other papers (e.g., Minder et al. 2018, Loh et al. 2022) covering much of the observations made in this study limit the scientific contribution in this work. Please find more specific comments below that hopefully guide the authors to improve the quality of this work.

Major comments

Introduction section does not give a sufficient literature overview regarding the limitations of subjective measures and potential pros and cons of the objective measures. Authors give a list of possible objective markers but it is difficult to get a grasp what criteria would have to be met that such a measure could be considered for a clinical use. Author's, for instance, note the existence of CPT test but do not acknowledge the decades of discussion whether it qualifies as a behavioural marker (e.g., Gualtieri & Johnson 2005). They also give classification accuracy values for a single study (Emser et al. 2018), although there are already previous meta-analyses giving much more comprehensive picture of the situation (e.g., Loh et al. 2022, Wang et al. 2022). Also the motivation for the concept naturalistic settings does not become clear from the introduction.

Results, especially the massive tables, are very difficult to digest. More careful synthesis of the findings would be appreciated. Especially concerning validity and reliability of the conducted studies. Also some comparison of the methods would be highly interesting. Also the discussion of the findings is rather superficial, which obviously follows the level of description in the results section. It maybe worth reporting e.g. where the research in this field has been done and how many studies for each topic have been conducted, but in the end more in depth analysis of the clinical value of OMs would probably be more important for the research community.

I appreciate that the study protocol was apparently preregistered. However, the document at their university is dated 21 September, 2022 and the text reads "…literature sources will be searched between 1st December 2021 and 28th February 2022 to identify papers relevant for inclusion." Which makes me doubt if this was actually a preregistration after all. This leads to another serious doubt. Since late 2021, a considerable amount of new literature has been published on this topic raising the question if the search should be updated.

Minor comments

Authors could reduce the number of unconventional abbreviations (e.g. starting from the abstract OM, CYP, NSs), it makes the paper quite heavy to follow.

Results and conclusions described in the abstract are not very informative.

"Objective measures have been suggested to be a valuable addition to the assessment process and were able to predict diagnosis with 78% accuracy alone (sensitivity = .80, specificity = .77, $\chi^2 = 17.09$, $p < .0001$), and in combination with the subjective measures with 86.7% accuracy (sensitivity = .83, specificity = .90, $\chi^2 = 29.53$, p <

VERSION 1 – AUTHOR RESPONSE

**Reviewer's comments**	
Interesting scoping review. Review and ensure unit reporting consistencies. For example, in some sections (Results and Characteristics of sources, some data is reported with n units and some with or without percentages).	Thank you for highlighting this. We have used the number of studies now rather than switching between units.
Methods - Descriptive statistics were used to analyses included papers (were descriptive statistics used on the 2801 papers identified or the 128 included studies). This makes it sound like methods were used on the 2801 larger n than 128.	Thank you for identifying this, we have updated the wording to reflect that the descriptive statistics refer to the 128 papers.
Major comments	
**Introduction section does not give a sufficient literature overview regarding the limitations of subjective measures and potential pros and cons of the objective measures.** Authors give a list of possible objective markers but it is difficult to get a grasp what criteria would have to be met that such a measure could be considered for a clinical use. Author's, for instance, note the existence of CPT test but do not acknowledge the decades of discussion whether it qualifies as a behavioural marker (e.g., Gualtieri & Johnson 2005). They also give classification accuracy values for a single study (Emser et al. 2018), **although there are already previous meta-analyses giving much more comprehensive picture of the situation (e.g., Loh et al. 2022, Wang et al. 2022)**. Also the **motivation for the concept naturalistic settings does not become clear from the introduction.**	Thank you for pointing this out. We have expanded on the strengths and limitations of objective measures in the introduction, as well as limitations of subjective measures. We have not gone into detail about CPTs as they lack naturalistic relevance within the scope of this review. Nonetheless, their objectivity warrants acknowledgment. We have now incorporated the reviews into the introduction, and written a more detailed explanation of the naturalistic focus. We have not included here the exact wording of the changes as they are lengthy and can be seen in the manuscript.
Results, especially the massive tables, are very difficult to digest.	Thank you for this appraisal, we have made substantial changes to the tables included in this review. We recognise that the size of tables were difficult to digest, we have made amendments to all tables in order to make them more manageable. We have split the larger tables (originally table 2 and 4) into two; with one containing data on observational measures and the other acceleration sensitive devices. Next, we have summarised phi coefficients, Pearson correlation, mean reliability coefficient, and K value in Tables 2 and 3 into one column. This has allowed us to keep the data, while removing some of the unwieldiness referenced by the reviewer.

More careful synthesis of the findings would be appreciated. Especially concerning validity and reliability of the conducted studies.	Thank you for this comment. The aim of the review is to assess the breadth of measures researched, we do not think this was explicitly clear and have now revised the aim to manage expectations. Synthesising validity and reliability is therefore beyond the scope of this scoping review. The research questions reference these descriptive aims, and .We have added more detail in the discussion section regarding the lack of reporting of psychometric properties in the studies reviewed, and have signposted a recently published reporting guideline.
Also some comparison of the methods would be highly interesting.	We have adjusted the wording of the "Characteristics of sources of evidence" section to be more descriptive of the aims of the included studies. We have also added a paragraph to the discussion to further explore this: "The papers primarily aimed to refine assessment methods and assess the efficacy of interventions, particularly school-based cognitive behavioural therapy programmes, often integrating objective measures alongside conventional assessment methods. This is reflective of best practice recommendations in literature, such as Emser and Hall (28,47), who highlight the added value of using an objective measure in ADHD assessment, as well as being reflective of clinical guidelines. Despite this, these are less used in clinical practice. This could be for numerous reasons, one being that the psychometric properties of one objective measure are reported to vary widely across studies, as seen in this review. Clinician and researcher confidence that objective measures capture change robustly may be impacted by this, leading to objective measures being used as an adjunct rather than a primary outcome. This review shows that there are, however, objective measures that are psychometrically sound, such as the BOSS; there remains a gap between research findings and real-world application, highlighting the need for further bridging of this divide to improve clinical outcomes. "
Also the discussion of the findings is rather superficial, which obviously follows the level of description in the results section. It maybe worth reporting e.g. where the research in this field has been done and how many studies for each topic have been conducted,	Thank you for identifying this, we have added more detail to the discussion regarding comparison of methods, lack of psychometric reporting, and clinical recommendations.
More in depth analysis of the clinical value of OMs would probably be more important for the research community.	Thank you for pointing this out. We have now added a paragraph to the discussion about the clinical value of objective measures.

I appreciate that the study protocol was *apparently preregistered.* However, the document at their university is dated 21 September, 2022 and the text reads "…literature sources will be searched between 1st December 2021 and 28th February 2022 to identify papers relevant for inclusion." Which makes me doubt if this was actually a preregistration after all. This leads to another serious doubt. Since late 2021, a considerable amount of new literature has been published on this topic **raising the question if the search should be updated.**	Thank you for this comment. The study protocol was finalised in February 2022 (the same dates as the searches were being finalised), however there was a delay in uploading to our institutional repository as it was neither a dataset nor an accepted manuscript (apparently our institution struggles to handle anything outside of this remit!). Systematic review protocols registered on prospero only need be registered before screening is complete, so we were ahead of the generally-accepted cutoff in the study process regarding protocol finalisation. We have revised the wording to "registered" in the manuscript to reflect this.<br><br>Whilst we appreciate that additional studies will have been published since the search dates, we argue that updating the searches is not necessary for the scoping review to have met it's aims. The searches were completed in Feb 2022, the screening, extraction, and analysis took approximately 12 months, and preparing the manuscript took us to September 2023 when this was submitted to the journal. This work forms part of a PhD and so was conducted by the lead author with the support of a supervisory team, alongside planning and delivering the other studies for their thesis. There is therefore no resource for us to update the searches at this time. We have added this to the limitations of the paper, "However, the searches were conducted in 2022, and publication in 2024 therefore means there may have been further relevant studies published that are not captured within our findings"
Minor comments	
Authors could **reduce the number of unconventional abbreviations** (e.g. starting from the abstract OM, CYP, NSs), it makes the paper quite heavy to follow.	Thank you for making us aware of this, the abbreviations did make the wording less coherent. We have removed OMs and NSs, however have kept CYP as it is quite commonly used in the field of child health.
Results and conclusions described in the abstract are not very informative.	Thank you for this comment. We have amended the results and conclusions of the abstract as much as possible within the word limit.
"Objective measures have been suggested to be a valuable addition to the assessment process and were able to predict diagnosis with 78% accuracy alone (sensitivity = .80, specificity = .77, $\chi 2 = 17.09$, $p < .0001$), and in combination with the subjective measures with 86.7% accuracy (sensitivity = .83, specificity = .90, $\chi 2 = 29.53$, $p < .0001$) (Emser et al., 2018)." -> Why give specific values from a single study as an example while there are tens of studies on this topic and the results are rather dependent on the experimental design and methodologies (I.e. findings are rather heterogenous, see Loh et al. 2022).	Thank you for identifying this, we have now made it more clear that psychometric properties can vary greatly regarding objective measures. We have added "Implementation could offer further evidence towards assessment. (29) Previous reviews have reported that objective measures can exhibit high reliability and validity but demonstrate variability. (32–34) For example, Minder et al (32) found the inter-rater reliability across systematic behavioural observations ranged from 0.61- 1 (Pearson's r) and from 0.39 to 0.99 (kappa coefficient), and convergent validity varied across studies and tools, with correlations ranging from poor to strong. Objective measures have been found to have good discriminant validity between ADHD

	and neurotypical people, however, are not as effective as discriminating between ADHD and other disorders. (32,35)”
”Sleep wasn't considered to be a core symptom.” -> Indeed, it is not a symptom of ADHD based on the diagnostic criteria. Please remove.	This sentence has been removed.
”Data charting was completed using a data charting tool developed by the lead author in Microsoft Excel.” -> Unclear	Thank you for pointing this out, we have changed the wording in the methods to reflect that excel was used to facilitate the process rather than a specific tool in excel was used.
”Papers were grouped and synthesised based on commonalities (e.g., type of OM, age group tested, core symptom tested).” -> Unclear how this was done and how the criteria were defined. Please specify.	Thank you for identifying this. We have added a description, following the sentence identified: “In the process of reviewing the included studies, we identified recurring themes and categories, which were prevalent across multiple papers. Consequently, we adopted these categories as the basis for our analytical framework. These categories included: title of paper, lead author, year of publication, journal taken from, country of origin, diagnostic status, type of diagnosis (if applicable) (e.g. Research or clinical), sub-types of diagnosis, eligibility criteria, total no. Of participants recruited, total no. Of participants with data, population age (mean and sd/range), gender [% male (1dp)], ethnicity (%), study design, aim. Some categories were specific for reviews: type of review, no. Of databases searched, names of databases searched, date range of included studies, additional searches, dates searches conducted, last updated search, total no. of participants, eligibility criteria for review”
Page 9, lines 36-37: ”Although the psychological response currently cannot be measured…” -> The sentence is unclear, please revise.	Thank you for pointing this out, we have removed this sentence.
Page 9, line 44: ”One type of psychophysiological measure common in our review was actigraphy.” -> I think it is misleading to consider actigraphy as a psychophysiological measure. Please explain or revise.	Thank you for pointing this out, although having read papers where actigraphy is categorised as a psychophsyiolocal measure, we can see how the definition of psychophysiology could create some issues when considering actigraphy. In line with Wang et al (2022), we have referred to actigraphs under the category "acceleration-sensitive measures" throughout the paper now.
Page 10, lines 51-52: ”Given the increasing interest in OMs, this review aimed to understand the range and types of OMs of Attention-Deficit/ Hyperactivity Disorder (ADHD) relevant to CYP which could be applied in NSs.” -> No need to redefine the abbreviation for ADHD here.	Thank you for identifying this, this has been adjusted accordingly.
Page 10, line 53:”The review found 90 Oms” -> …OMs	Thank you for identifying this, this has been adjusted accordingly.
Page 11, line 43: ”This is especially true where children with SEN” -> I missed if the abbreviation	Thank you for making us aware of this, we have now added "special educational needs" before

for SEN is explained somewhere.	SEN

VERSION 2 – REVIEW

**REVIEWER NAME**	***Salmi , Juha***
**REVIEWER AFFILIATION**	University of Turku
**REVIEWER CONFLICT OF INTEREST**	Na
**DATE REVIEW RETURNED**	22-May-2024

GENERAL COMMENTS	General comment<br><br>Manuscript by Kelman and colleagues is a scoping review examining the opportunities of objective measures in the diagnostic assessment of ADHD. The authors have revised the manuscript accounting for several of my comments. However, some issues still remain.<br><br><br>Major comments<br><br>There is a considerable amount of typos in the manuscript, some probably related to heavy editing with track changes option. Due to the large number of typos, I will not specify each of them but trust that the next version of the manuscript is improved in quality. I would nevertheless like to note that it makes reviewer's work a bit frustrating when the font type and size as well as spacing is changing wildly for several times, and several sentences end in weird way etc.<br><br>It does not make sense to include Percentage agreement, Kappa, Other Value, and Test-retest for studies using acceleration-sensitive devices. Please extract more some meaningful data from those studies (e.g., classification accuracies, group comparison stats). Overall, it seems that although many of the studies do not report Sensitivity / Specificity they do report other quantitative data that would be valuable to report (group stats, AUC etc.).<br><br>Minor comments<br><br>Abstract:<br><br>Embase, (via OVID) -> Embase (via OVID)<br><br>MEDLINE...we searched between 1st December 2021 and 28th February 2022. -> Please revise<br><br>There is a lack of reporting of psychometric properties and guidance for researchers administering these measures in practice and in future studies. -> In general, I do agree with this statement. However, there is a recent study by Jakov et al. (https://link.springer.com/chapter/10.1007/978-3-031-59091-7_12) that focused exactly on this (and also assessed diagnostic accuracy - see the previous sentence).<br><br>Table 2 -> why some lines in First author section have a number and not the last name of the first author? Pr = Pearson's correlation coefficient (?)

VERSION 2 – AUTHOR RESPONSE

Reviewer: 2	
Dr. Juha Salmi , University of Turku	

Comments to the Author:	
Manuscript by Kelman and colleagues is a scoping review examining the opportunities of objective measures in the diagnostic assessment of ADHD. The authors have revised the manuscript accounting for several of my comments. However, some issues still remain.	Thank you for taking the time to review our paper. We appreciate your thoughtful feedback and the opportunity to further improve our work.We believe these revisions have strengthened the manuscript and addressed the remaining concerns.
Major comments	
There is a **considerable amount of typos in the manuscript**, some probably related to heavy editing with track changes option. Due to the large number of typos, I will not specify each of them but trust that the next version of the manuscript is improved in quality. I would nevertheless like to note that it makes reviewer's work a bit frustrating when the font type and size as well as spacing is changing wildly for several times, and several sentences end in weird way etc.	Thank you for identifying this, we appreciate that is frustrating. We have had the script proofread to ensure the manuscript does not have typos, change font type, or size, or change spacing.
It does not make sense to include Percentage agreement, Kappa, Other Value, and Test-retest for studies using acceleration-sensitive devices. Please extract more some meaningful data from those studies (e.g., classification accuracies, group comparison stats). Overall, it seems that although many of the studies do not report Sensitivity / Specificity they do report other quantitative data that would be valuable to report (group stats, AUC etc.).	Thank you for pointing this out, wehave rescreened the papers to check for and extract more meaningful, especially data regarding acceleration-sensitive devices (e.g. group comparisons and AUC). Where papers only used correlation coefficients to quantify agreement, statistics were not reported. Group comparisons were reported when comparing ADHD groups to control groups.These have been captured in Table 3.
Minor comments	
Abstract:	
Embase, (via OVID) -> Embase (via OVID)	Thank you for highlighting this, we have removed the comma.
MEDLINE...we searched between 1st December 2021 and 28th February 2022. -> Please revise	Thank you for identifying this, the spelling has been revised.
There is a lack of reporting of psychometric properties and guidance for researchers administering these measures in practice and in future studies. -> In general, I do agree with this statement. However, there is a recent study by Jakov et al. (https://link.springer.com/chapter/10.1007/978-3-031-59091-7_12) that focused exactly on this (and also assessed diagnostic accuracy - see the previous sentence).	Thank you for highlighting this and signposting us to this study, we have added to the comment in the abstract to "encouragingly, recent papers begin to address these issues." We have also added an amendment to the discussion, “Encouragingly, recent papers begin to address these issues. For example, Basic et al (160) explored the use of motion sensors for detecting ADHD and found high accuracy

	with advanced computational methods. However, they emphasise a need for further validation and integration with other methods.”
Table 2 -> why some lines in First author section have a number and not the last name of the first author? Pr = Pearson's correlation coefficient (?)	Thank you for identifying this, we have made amendments to Table 2 and changed Pr to r.

VERSION 3 – REVIEW

**REVIEWER NAME**	***Salmi , Juha***
**REVIEWER AFFILIATION**	University of Turku
**REVIEWER CONFLICT OF INTEREST**	Na
**DATE REVIEW RETURNED**	15-Jul-2024

**GENERAL COMMENTS**	I have no remaining comments.
